# Supplementary material for: Safety and immunogenicity of booster vaccination and fractional dosing with Ad26.COV2.S or BNT162b2 in Ad26.COV2.S-vaccinated participants
Source: PLOS Glob Public Health. 2024 Apr 11;4(4):e0002703. doi: 10.1371/journal.pgph.0002703 (PMC11008839; doi:10.1371/journal.pgph.0002703)
Supplement: S1 Protocol — (PDF) [file pgph.0002703.s001.pdf]

**Trial Title:**	Phase II randomised open label trial of full and half dose J&J Ad26.CoV2.S and Pfizer BNT162b2 booster vaccinations after receiving the J&J Ad26.CoV2.S prime vaccine through the SISONKE phase IIIB implementation study or through the South African COVID-19 vaccination programme.
**Study Reference:**	J&J Ad26.COV2.S/Pfizer BNT162b2 (mRNA) prime-boost study<br><br>BaSiS study (**B**ooster **a**fter **Si**sonke **S**tudy)
**Protocol Version:**	Version 3.0
**Date:**	10 November 2022
**Protocol Chair**:<br>**Co-Chairs**:	Lee Fairlie (Wits RHI)<br>Penny Moore (NICD and University of the Witwatersrand)<br>Alex Sigal (AHRI)<br>Wendy Burgers (UCT)
**Sponsor:**	South African Medical Research Council<br>South African Department of Health (DoH)<br>Bill and Melinda Gates Foundation (BMGF)
**Funder:**	South African Medical Research Council<br>Bill and Melinda Gates Foundation (BMGF)

Table of Contents

1. Study Team 4
2. Abbreviations 6
3.0. Synopsis 10
4. Background and rationale 13
 4.1.Background 13
  4.1. 1. J&J Ad26.COV2.S 13
  4.1.2. Pfizer BNT162b2 vaccine 15
  4.1.3. Immune response in people living with HIV and other comorbidities 15
  4.1.4. Homologous and Heterologous boost studies 16
  4.1.5. Fractional dosing of vaccines 17
 4.2. Rationale for the primary study: 18
 4.3. Objectives and endpoint measures for BaSiS Primary study 18
 4.4. Progress on BaSiS Primary Study 20
 4.5. BaSiS Primary study Interim results 22
5. BaSiS Extension study 22
 5.1. Rationale for extension of study to 12, 18 and 24 month visits: 23
  5.1.1. Long term follow-up of BaSiS participants 23
  5.1.2. Rationale for HLA typing of all participants 23
  5.1.3. Rationale for isolating and characterizing the infecting virus from breakthrough infections (BTI) 24
5.2. Approach per extension study objective 24
  5.2.1. Objective 1. Long term follow-up of the BaSiS cohort 24
  5.2.2. Objective 2. Monitor long-term humoral immunity in PLHIV after vaccine boosting 26
  5.2.3. Objective 3. Characterize SARS-CoV-2 viruses in breakthrough infections 27
  5.2.4. Objective 4. Monitor long term immunological memory in PLHIV 28
  5.2.5. Objective 5. Perform HLA typing of the cohort to understand T cell breadth, specificity and escape potential 29
6. Methodology 33
 6.1. Trial design 33
 6.2. Study groups for the extension study 33
 6.3. Trial participants 34
 6.4. Potential risks for participants 34
  6.4.3. Venipuncture 34
  6.4.4. Vaccine reactions to additional booster vaccines 34
  6.4.5. Response to booster 35
  6.4.6. Anaphylactic reactions 35
  6.4.7. Loss of privacy or confidentiality 35
 6.5. Known potential benefits 35
 6.6. Recruitment and Withdrawal of trial participants 35
 6.7. Informed consent process 35
 6.8. Inclusion and exclusion criteria 36
  6.8.1. Inclusion criteria 36
  6.8.2. Exclusion criteria 37
  6.8.3. Female participants of childbearing potential 37
 6.9. Trial procedures 37
  6.9.1. Observations 37
  6.9.2. Spike binding antibody assays 37
  6.9.3. Neutralisation Assays 38

6.9.4. T cell assays 38
6.10. Study visits 39
6.10.1. Screening and enrolment visit (Day 0), 2-week, 3-month and 6-month visits (Participants off study) 39
6.10.2. Month 6 visit (±14 days) (Participants still on study) 39
6.10.3. Repeat booster visit (6 months +4 months) (Participants completed study) 39
6.10.4. Month 12 visit (Participants completed study) 39
6.10.5. Month 18 visit 40
6.10.6. Month 24 visit 40
6.10.7. Booster vaccination visit 12-24 months 41
6.10.8. Interim/Illness visits - only if indicated 41
7. Investigational Product 43
7.1. J&J Ad26.COV2.S adenovector vaccine 43
7.2. Pfizer BioNTech BNT162b2 modRNA vaccine 43
7.3. The Covovax™ vaccine 44
7.4. IP preparation, handling, storage and accountability 44
7.4.1. J&J Ad26.COV2.S adenovector vaccine 44
7.4.2. Pfizer BioNTech BNT162b2 modRNA vaccine 44
7.5. IP destruction 45
8. Assessment of safety 46
8.1. Definitions 46
8.1.1. Adverse Event (AE) 46
8.1.2. Adverse reaction (AR) 46
8.1.3. Serious Adverse event (SAE) 46
8.1.4. Important medically attended event 46
8.1.5. Suspected Unexpected Severe Adverse Reaction (SUSAR) 47
8.1.6. Adverse Event of Special Interest (AESI) 47
8.2. Grading of events 47
8.2.1. Reactogenicity 47
8.2.2. Vital signs 48
8.3. Causality 49
8.4. COVID BTI's will be graded as described below. 50
8.5. Reporting of AEs 50
9. Data Management and Analysis 51
9.1. Randomisation Procedure 51
9.2. Sample size 51
9.3. Database development 52
9.4. Data Entry and Management 52
9.4.1. Study-related Forms 52
9.4.2. Data Entry and Processing 53
9.4.3. Data Security 53
9.4.4. Data Quality Control 53
9.4.5. Data Transfer 54
9.5. Data Analysis 54
9.5.1. Immunogenicity analyses 55
9.5.2. Safety and Reactogenicity analyses 56
10. References 57
11. Appendices 63

1. Study Team

Table 1: Protocol team roster

South African PI and co-chair	Assoc. Professor Lee Fairlie<br>Wits RHI Shandukani<br>7 Esselen street<br>Hillbrow, 2001<br>Phone: +27827809997<br>Email: lfairlie@wrhi.ac.za
Co-chairs	Professor Penny Moore<br>HIV Virology Section, National Institute for Communicable Diseases, 1 Modderfontein Road, Johannesburg, South Africa<br>Phone: +27 72 72 44501<br>Email: pennym@nicd.ac.za
	Assoc. Professor Alex Sigal<br>Africa Health Institute and University of KwaZulu-Natal<br>719 Umbilo Road, Durban, South Africa<br>Phone: +27 83 446 3092<br>Email: alex.sigal@ahri.org
	Professor Wendy Burgers<br>Room 3.36.1, Level 3, Falmouth Building, Faculty of Health Sciences; University of Cape Town, 1 Anzio Road, Observatory, 7925, Cape Town, South Africa<br>Phone: +27825109071<br>Email: wendy.burgers@uct.ac.za
Sponsor	Professor Glenda Gray<br>SA MRC<br>Department of Health, South Africa
Funder	SAMRC, BMG
Trial sites	Wits RHI Shandukani Research Centre<br>7 Esselen Street, Hillbrow Johannesburg, South Africa, Gauteng, 2001<br>Phone: +27 11 358 5502 Fax: +27 86 548 4889<br>Email: lfairlie@wrhi.ac.za<br>Site PI: Dr Faeezah Patel: fpatel@wrhi.ac.za
	PHRU Kliptown<br>66 Sailor Malan Drive, Ground Floor, Omnipark, Block 1, Aeroton, Johannesburg South, Gauteng, South Africa, 2013<br>Tel: 011 342 4075<br>Email: lazaruse@phru.co.za; nanaa@phru.co.za<br>Site Co-PIs:<br>Clinical Co-PI: Dr Erica Lazarus<br>Non-clinical Co-PI: Anusha Nana
	CAPRISA eThekwini Clinical Research Site<br>No. 3 Richards Road, Warwick Avenue, Berea, Durban, 4001, KwaZulu-Natal, South Africa<br>Phone: +27 655 0613<br>Email: nigel.garrett@caprisa.org<br>Site PI: Dr Nigel Garrett
	Desmond Tutu Health Foundation: Masiphumelele CRS

	3 Guinea Fowl Road<br>Sunnydale, Cape Town<br>7975<br>Tel: 021 7853121<br>Email: Katherine.gill@hiv-research.org.za<br>Site PI: Dr Katherine Gill
**Co-Investigator**	Professor Helen Rees<br>Executive Director, Wits RHI<br>Corner Esselen and Klein Street<br>Hillbrow, 2001<br>Phone: 011 358 5300<br>Email: hrees@wrhi.ac.za

2. Abbreviations

ACLS - Advanced Cardiovascular Life Support

AE - Adverse Event

AR - Adverse Reaction

ART - Antiretroviral Treatment

AESI - Adverse Event of Special Interest

BCEPS – Biometric Co-enrolment Prevention System

BHCG - Beta Human Chorionic Gonadotropin

CAPRISA - Centre for the AIDS Programme in South Africa

ChAd - Chimpanzee Adenovirus

COVID-19 - Coronavirus Disease 2019

FDA - Food and Drug Administration

HCW - Health care worker

HIV - Human Immunodeficiency Virus

HLA – Human Leukocyte Antigen

ICF - Informed consent form

IPV - Inactivated Polio Vaccine

LICs – Low-income countries

LVNA - live virus neutralisation assay

mAbs - monoclonal antibodies

mRNA - messenger Ribonucleic Acid

nAbs- Neutralising Antibodies

NICD - National Institute for Communicable Diseases

PBMC - Peripheral Blood Mononuclear Cells

PBS - Phosphate-Buffered Saline

PCR - Polymerase Chain Reaction

PHRU - Perinatal HIV Research Unit

PLHIV - People Living with HIV

PNA - Pseudovirus Neutralisation Assay

POC - Point of care

SAE - Serious Adverse Event

SMS - Short Message Service

SUSAR - Suspected Unexpected Serious Adverse Reaction

TMB - Tetramethylbenzidine

UK - United Kingdom

VOC - Variant of concern

VL - Viral Load

Wits RHI - Wits Reproductive Health and HIV Institute

WOCP - Women of childbearing potential

Table 2: Summary of changes for Version 3.0

	Version 2.0 dated 23 March 2022	Version 3.0 dated 10 November 2022
1.	SA MRC as funder	Addition of Bill and Melinda Gates foundation as funder
2.	Original chairs listed (Fairlie, Moore, Sigal)	Addition of Prof Wendy Burgers as co-chair
3.	Study visits baseline, 2-weeks, 3-months and 6-months	Extension of primary study to include visits at 12-, 18 and 24 months
4.	Randomisation approach discussed	Updated information regarding the randomisation arms in the primary BaSiS study including number or participants per arm, HIV status, age. (Table 4, 5)
5.	Analysis plan describes interim analysis and intention to vaccinate participants with < 75% geometric mean titres compared to best arm and individual response	Description of the interim analysis and DSMB recommendation for additional Pfizer BNT162b2 vaccination for most participants based on the analysis
6.	Pfizer booster vaccination provided based on interim analysis	Discussion of additional extension booster on extension study. Options are discussed including a repeat Pfizer BNT162b2, a Novovax/Covovax booster or a bivalent vaccine. Finalisation will occur based on participant data, available vaccines and VOC
7.	BaSiS Primary study objectives listed	Extension study objectives including:<br>• Long term follow-up of the BaSIS cohort including additional visits at 12 months, 18 months and 24 months compared to baseline.<br>• Monitor long-term humoral immunity in PLHIV after vaccine boosting<br>• Characterize SARS-CoV-2 viruses in breakthrough infections<br>• Monitor long term immunological memory in PLHIV<br>• Perform HLA typing of the cohort to understand T cell breadth, specificity and escape potential
8.	Original background and rationale updated for version 2.0	Background and rationale section updated
9.	BaSiS Primary study design and analysis plan discussed	Progress on BaSiS Primary study discussed as well as interim results
10.	BaSiS primary study objectives and endpoints table 3	Detailed description of the extension study including objectives and endpoints
11.	BaSiS Primary study laboratory sections	Detailed description of laboratory-testing components under each extension objective
12.	BaSiS primary study SoE included in Table 6 unbolded	Revised schedule of evaluations with extension study bolded (Table 6)
13.	Methodology for primary study	Methodology revised for the extension study

14.	No HLA testing	Addition of HLA testing for participants and HLA-testing ICF
15.	Safety and reactogenicity primary objective	Revision of safety evaluation to exclude diary cards for the extension study, given the millions of people vaccinated for COVID-19 and restricting safety evaluations as tabulated (Table 6)
16.	Visit procedures for primary study	Updated visit procedures for the extension visits 12-, 18- and 24-months
17.	J&J Ad26.COV2.S and Pfizer BNT162b2 descriptions	Updated product description by adding Novovax as a potential heterologous booster on the extension study (extension booster)
18.	BaSiS Primary study data sections	Data sections updated

3.0. Synopsis

Title	Phase II randomised open label trial of full and half dose J&J Ad26.COV2.S and Pfizer BNT162b2 booster vaccinations after receiving the J&J Ad26.COV2.S prime vaccine through the SISONKE phase IIIB implementation study or through the South African COVID-19 vaccination programme-Extension
Short title	J&J Ad26.COV2.S/Pfizer BNT162b2 prime-boost study
Study name	BaSiS study (**B**ooster **a**fter **Si**sonke **S**tudy)
Sponsor	South African Medical Research Council (SA MRC)<br>South African Department of Health (DoH)<br>Bill and Melinda Gates Foundation (BMGF)
Funder	SA MRC<br>BMGF
Trial design	Open label randomised phase II study of immunogenicity after one of four booster Covid-19 vaccine doses with the J&J Ad26.COV2.S (full or half dose) or the Pfizer BNT162b2 (full or half dose) vaccine, received after an initial J&J Ad26.COV2.S prime via the SISONKE phase IIIB implementation study or through the South African COVID-19 vaccination programme. Initial study follow-up until 6 months post-booster is being extended to 24 months post-booster.
Trial participants	This extension study builds on the primary BaSiS study, where participants who received a single dose of a J&J Ad26.COV2.S vaccine were recruited from the SISONKE phase IIIB implementation study and through the South African COVID-19 vaccination programme. Participants were eligible for enrolment if they were 18 years of age and older and we aimed to recruit at least 30 % of people living with HIV (PLHIV) and 10% of participants ≥ 55 years of age. Participants received the prime J&J Ad26.COV2.S vaccine at least 4 months prior to enrolment in the BaSiS study. Participants who had previously been infected with SARS-CoV-2 (at least 28 days post symptom resolution or post positive test in asymptomatic) and participants with well controlled comorbidities were enrolled in the study. In total 289 vaccinated participants were enrolled, with randomisations as follows (Table 4, 5:<br>**Group A** = 74 Ad26.COV2.S prime plus full dose Ad26.COV2.S booster (50 HIV- 25 HIV+) at a dose of 5x 10^{10} vp/ ml (0.25 ml)<br>**Group B** = 69 Ad26.COV2.S prime plus half dose Ad26.COV2.S booster (50 HIV- 25 HIV+) at a dose of 2.6x 10^{10} vp/ ml (0.13 ml)<br>**Group C** = 73 Ad26.COV2.S prime plus full dose BNT162b2 booster (50 HIV-, 25 HIV+) at a dose of 30mcg (0.3 ml)<br>**Group D** = 73 Ad26.COV2.S prime plus half dose BNT162b2 booster (50 HIV-, 25 HIV+) at a dose of 15mcg (0.15 ml)<br>The subgroups included in the study as follows- (Table 4):<br>1) 173 (60%) participants HIV-uninfected<br>2) 116 (40%)participants are PLHIV<br>3) 36 (12%) participants ≥ 55 years

	Per site: Wits RHI – 102 enrolled; CAPRISA – 27 enrolled; PHRU- 56 enrolled; Masiphumelele – 104 enrolled.
Sample size	Enrolment is complete as above and participants are in follow-up. The extension study will follow up participants at additional 12-month, 18-month and 24-month visits post the initial BaSiS booster vaccination.
Planned trial period	Study enrolment was completed on the 29th July 2022. The primary study had follow-up visits at 2 weeks, 3 months, and 6 months (study exit) from the enrolment date. The extension study has additional 12-month, 18-month and 24-month visits.<br>In addition, interim visits are arranged for any solicited adverse events (AEs) beyond 7 days post vaccination, or unsolicited AEs throughout the study post vaccination if they are grade 3 or higher.<br>Additional study visits are conducted for participants who develop symptoms of SARS-CoV-2 infection or test positive for SARS-CoV-2 infection with a nasopharyngeal swab polymerase chain reaction test or rapid antigen test while on the BaSiS primary study.
Interim analysis	An interim analysis for immunogenicity was performed after the 3-month visit. The highest geometric mean titre (GMT) of neutralising antibodies, calculated per arm for each of the four arms, was used as the benchmark for comparison to individual immunogenicity responses. The GMT of neutralising antibodies from the 2-week and 3-month post-vaccination visit was utilised for this purpose. As written in version 2.0 of the protocol, if insufficient immunogenicity (defined as <75% of the GMT) was observed between the full dose and half dose arms, or between J&J Ad26.COV2.S and Pfizer BNT162b2 arms, regardless of dose, the homologous or heterologous booster vaccine eliciting the most robust response will be offered.<br>Interim data including analyses from the 2-week and 3-month visits, showed a more robust antibody and cellular immune response with the Pfizer full dose vaccine, compared to the J&J full and half dose vaccines, as well as the Pfizer half dose vaccine, all of which were <75% GMT of the Pfizer response.<br>On the 24th August 2022, the study Data and Safety Monitoring Board (DSMB) reviewed the data and recommended that all participants who received any of these boosters, except for the full dose Pfizer, receive a full dose Pfizer boost. People who received a full dose Pfizer vaccine on BaSiS >6 months prior were also offered a repeat full dose Pfizer boost in line with South African recommendations.<br>In the extension study, information regarding uptake of this additional Pfizer boost or any other vaccination and breakthrough infection will be collected.
Study objectives for the extension study	• Long term follow-up of the BaSIS cohort including additional visits at 12 months, 18 months and 24 months compared to baseline.<br>• Monitor long-term humoral immunity in PLHIV after vaccine boosting<br>• Characterize SARS-CoV-2 viruses in breakthrough infections<br>• Monitor long term immunological memory in PLHIV

	• Perform HLA typing of the cohort to understand T cell breadth, specificity and escape potential
Study procedures	Based on the interim analysis and DSMB recommendation, as discussed in "interim analysis" all participants who received a J&J Ad26.COV2.S booster (half or full dose) as well as a Pfizer BNT162b2 half dose booster, were offered an additional Pfizer BNT162b2 booster, as well as people on BaSiS who had received a Pfizer BNT162b2 full dose >6 months prior.<br>For the extension study we will explore the possibility of giving participants an additional "extension" booster based on available data regarding variants of concern, available vaccines and participant data on the extension study. Additional clarity will be provided in a clarification memo.

4. Background and rationale

4.1.Background

In March 2020 a global pandemic of COVID-19 caused by the SARS-CoV-2 virus was declared, and subsequently over 650 million people have been confirmed as SARS-CoV-2 positive and over 6.5 million deaths have been directly attributed to COVID-19 globally, with 4 million cases and over 100, 000 deaths reported in South Africa (1). The ancestral SARS-CoV-2 strain has mutated over time and variants of concern (VOC) have emerged including the B.1.1.7 (Alpha), B1.351 (Beta), B.1.617.2 (Delta) and P.1 (Gamma) variants (2) and more recently the B.1.1.529 BA.1,BA.2, BA.4 and BA.5 (Omicron) variants identified in November 2021 and through 2022 in South Africa (3). In South Africa, the beta variant was predominant in the second wave of infection, and the delta variant was dominant in the third wave, with both variants known to have increased transmissibility and escape from neutralisation by convalescent and vaccine induced antibody immunity. The delta variant appears to result in more severe disease relative to the alpha variant, with data from Scotland reporting a risk of hospitalisation with delta variant infection double that of the alpha variant (4). In pregnant women, alpha and delta variant strains result in increased severity of maternal disease and adverse pregnancy outcomes compared to the ancestral strain (5). The omicron variant, largely responsible for the fourth wave, displayed high transmissibility but apparently milder disease with lower rates of severe disease, hospitalisation and death (6, 7). However, Omicron-triggered neutralisation in unvaccinated SARS-CoV-2 naïve individuals is not extensively cross-reactive to VOC, leaving these individuals likely to be more susceptible to reinfection by circulating and emerging VOC that may potentially be more virulent(8). Throughout 2020, tremendous efforts to develop effective, safe COVID-19 vaccines resulted in emergency use authorization of vaccines with different mechanisms of action, and more recently licensure of some vaccines. Among these are the single dose Johnson & Johnson (J&J) adenoviral vectored J&J Ad26.COV2.S vaccine and the two-dose Pfizer BNT162b2 mRNA vaccines, both of which use the SARS-CoV-2 ancestral spike glycoprotein sequence as the antigen. Other vaccine types include inactivated virus vaccines (Vaxzevria/AstraZeneca/ChAdOx, CoronaVac, Covaxin and others), and protein subunit vaccines such as the Novavax NVX-CoV2373 (9). Globally, over 12 billion vaccine doses have been administered, almost 68% of the world's population have received at least one vaccine dose and just under 5 billion people have completed vaccination. However, in low-income countries, only 20.7% of people have been vaccinated (10), resulting in a need for strategies to accelerate vaccine coverage in the context of limited vaccine supplies and the high cost of vaccines. South Africa has expanded vaccine access in a stepwise manner: initially single dose J&J Ad26.COV2.S to health care workers then those with high exposure such as school teachers and the police force, followed by either the two-dose Pfizer BNT162b2 vaccine regimen or single dose J&J Ad26.COV2.S vaccine to those over 60 years of age, then to over 35-year-olds, then to all people over 18-years of age (from 1st of September 2021). In October 2021, the age of eligibility for Pfizer BNT162b2 was expanded to include 12-18 year olds. Since booster doses were introduced late December 2021 and since February 2022, homologous and heterologous booster vaccines have been made available for those previously vaccinated. Here we focus on the J&J Ad26.COV2.S and Pfizer BNT162b2, two of currently approved vaccines in South Africa.

4.1. 1. J&J Ad26.COV2.S

The J&J Ad26.COV2.S vaccine produced by Johnson & Johnson is a single dose, replication-incompetent, recombinant, adenovirus serotype 26 vectored SARS-CoV-2 spike protein vaccine. The safety and efficacy of the J&J Ad26.CoV.S was assessed globally in the ENSEMBLE phase 3 trial, a randomised,

placebo-controlled, double-blind study (11). The study enrolled 44 000 participants, almost 7000 from South Africa, and demonstrated an overall vaccine efficacy against moderate to severe disease of 66.9% (adjusted 95% confidence interval [CI], 59.0 to 73.4) (11). The vaccine had greater efficacy against severe and critical COVID-19 for those infected ≥14 days post vaccine, 76.7% (adjusted 95% CI, 54.6 to 89.1), for those with onset of disease ≥28 days post vaccine, 85.4% efficacy (adjusted 95% CI, 54.2 to 96.9)(11). In South Africa, during the Ensemble trial the majority (94.5%) of SARS-CoV-2 infections were due to the beta variant (20H/501Y.V2), and vaccine efficacy against infections ≥14 days after the dose was 52.0% and ≥28 days, 64.0%, against moderate-to-severe/critical SARS-CoV-2 infection. Against severe-critical infection, vaccine efficacy was 73.1% at ≥14 days and 81.7% at ≥28 days(11). As expected, reactogenicity was higher in the vaccine compared to placebo group but most events were mild to moderate and serious adverse events were distributed across arms, with no SARS-CoV-2 related deaths in the vaccine arm (11).

Following on from the J&J ENSEMBLE study, in South Africa approximately half a million health care workers received the single dose J&J Ad26.COV2.S vaccine through the SISONKE phase IIIB implementation study (12). Interim data from the study showed only around 2% of vaccinees reported any adverse events and that thrombocytopenic thromboembolism, initially reported with the ChAdOx1 nCoV-19 vaccination, also adenovirus based, was extremely rare and occurred in only 1.7/100 000 participants, (13). Recent data showed approximately 2% of HCW had breakthrough infection with SARS-CoV-2, mostly mild (95.6%), with 2.76% moderate, 0.49% severe and 0.4% resulting in death; 76% of people who died had comorbidities (12). The single J&J Ad26.COV2.S vaccine resulted in 65-66% protection against hospitalisation and 91-96% protection against death (12). These results were reported over the time of the beta and delta variants, suggesting a good response across both variants. In addition, at 8 months post vaccine, Barouch et al., report sustained durability and neutralising antibody breadth expansion over this time period, suggestion B-cell response maturation without additional boosting (14). Data from the J&J Ad26.COV2.S phase 1/2a study (COV1001) similarly show that neutralising antibodies remain stable until 8-9 months post prime, and binding antibodies until 6 months post prime, regardless of age (15). Additional doses of the J&J Ad26.COV2.S vaccine have been made available to South Africa and other sub-Saharan African countries.

Although the J&J Ad26.COV2.S vaccine as a single dose is compelling programmatically due to reduced costs, simpler storage requirements and less complex logistics, especially for health systems in resource constrained settings, the prevalence of breakthrough infections, although largely mild, indicated that protection against infection might need to be improved in Ad26.COV2.S vaccinees. In addition, maintaining protection from severe disease over time, in light of the emergence of VOC, is essential. Protection from infection correlates with high antibody titres elicited by vaccines (16) and reduced neutralisation titres have been reported for J&J Ad26.COV2.S vaccines against the beta, delta and omicron variants compared to the ancestral variant (17). This prompted the authors of these studies to suggest that a second J&J booster or a heterologous prime-boost strategy may enhance titres and reduce infections, and ensure ongoing and durable protection following immunisation (17). SISONKE-2 enrolled 227 310 HCW from the original SISONKE implementation study prior to the start of the Omicron fourth wave in South Africa. Participants were given a homologous boost of the J&J Ad26.COV.2.S vaccine 6-9 months after their initial vaccination. After adjusting for confounders, VE was shown to increase over time with 85% prevention of hospitalisation at 1-2 months post-boost (18). Given the benefits of booster vaccination in the context of inequity of vaccine access in Africa, understanding whether fractional dosing, further described in section 4.1.5, will produce similarly robust immune responses is critical.

4.1.2. Pfizer BNT162b2 vaccine

The Pfizer BNT162b2 vaccine, a messenger RNA (mRNA) vaccine which encodes the secreted trimerised SARS-CoV-2 spike protein, has also had extremely favourable results. In the phase I study, the Pfizer BNT162b1 and Pfizer BNT162b2 vaccines were evaluated at doses of 10mcg, 20mcg, 30mcg and 100mcg. Vaccines were administered 21 days apart, except the 100mcg dose which was a single dose, to evaluate safety and immunogenicity. The Pfizer BNT162b2 vaccine at a 30mcg dose was selected for further development, due to the slight decrease in reactogenicity compared to the Pfizer BNT162b1 vaccine and favourable immunogenic responses for SARS-CoV-2 neutralising antibody, although the 20mcg dose also performed well, with similar responses compared to 30mcg (19). The phase II/III multinational randomised placebo-controlled study enrolled over 43 000 participants and evaluated safety and efficacy, demonstrating a 95% efficacy and favourable safety profile for the Pfizer BNT162b2 vaccine (20). Globally hundreds of millions of people, over 200 million in the United States alone, 18 years and older have received this vaccine (21). Adverse events surveillance has reported anaphylaxis - 11.1 per million cases meeting Brighton Classification case definitions, and myocarditis - 40.7 per million cases, which has been reported more commonly in males less than 30 years of age (22, 23). Benefit risk analyses have been done and found that the risks of COVID-19 disease far outweigh the comparatively small risks of these adverse events (22, 23). A study evaluating the effectiveness of mRNA vaccines in HCWs including the Pfizer BNT162b2 and the Moderna vaccine in the United States, reported a vaccine efficacy of 82% (95%CI = 74%–87%) after a single dose and 94% (95% CI = 87%–97%) after 2 doses (24). "Real world" effectiveness data from national surveillance in Israel in the first four months of the Pfizer BNT162b2 vaccine rollout reported adjusted rates of effectiveness from 7 or more days after the second vaccine (25). They report 95.3% (95% CI 94.9–95.7) effectiveness overall, 91.5% (95% CI 90.7–92.2) effectiveness against asymptomatic SARS-CoV-2 infection; 97.0% (95% CI 96.7–97.2) for symptomatic COVID-19 infection; 97.2% (95% CI 96.8–97.5) for hospitalisation related to COVID-19 and 96.7% (95% CI 96.0–97.3) effectiveness against death related to COVID-19 (25). When evaluating effectiveness against variant strains, Bernal et al., reported a slight decrease in effectiveness in people vaccinated with two doses of the Pfizer BNT162b2 vaccine between the alpha [93.7% (95% CI, 91.6 to 95.3)] and delta variant [88.0% (95% CI, 85.3 to 90.1)] (26). This suggests a slight but insignificant loss in effectiveness with the delta variant, but still high levels of effectiveness against this variant (26). Immunogenicity data supports this high level of efficacy and effectiveness, with the Pfizer BNT162b2 vaccine eliciting extremely high titres against the original variant, and modest preservation of neutralisation activity against VOCs including beta and delta (17). In South Africa, data from the fourth omicron-driven COVID-19 wave showed a 70% (95% CI, 62 to 76) vaccine efficacy against the Pfizer BNT162b2 vaccine for hospitalisation, a reduction from 93% (95% CI, 90 to 94) in previous waves(27). Despite the number of spike protein mutations in the Omicron variant, increasing transmissibility and reduction in neutralising antibodies, T-cell responses to both Pfizer BNT162b2 and J&J Ad26.COV2.S appear maintained and similar to that seen in previous waves (28).

4.1.3. Immune response in people living with HIV and other comorbidities

South Africa has the largest HIV epidemic in the world, with 7.7 million people living with HIV (PLHIV) with an overall prevalence of about 19% and approximately 70% on antiretroviral therapy (ART) (29). Immunosuppression caused by HIV is known to interfere with protective vaccination against multiple pathogens, typically as a consequence of sub-optimal antibody responses (30-33).

In line with this, results from a South-African phase IIb trial of the Novavax NVX-CoV2373 vaccine, which uses a stabilised prefusion spike protein, showed 60% efficacy in HIV-uninfected individuals. However, overall efficacy dropped to 49% upon inclusion of PLHIV, although it is important to note that the

numbers of PLHIV in the study were very small (34). Nonetheless, there were more breakthrough SARS-CoV-2 infection cases in PLHIV in the vaccine arm than the placebo arm. A small study evaluating immune response in PLHIV compared to HIV-uninfected controls 7-17 days after the second Pfizer BNT162b2 vaccine showed similar spike protein binding antibody titres, neutralising antibody to the vaccine spike protein as well as variants of concern including the beta and delta variants (35). Cellular immune responses between the groups were similar (35). In South Africa, 104 PLHIV well controlled on ARVs, were enrolled in the ChAdOx1 nCoV-19 study and had comparable tolerability, safety and immunogenicity responses to the vaccine compared to HIV-uninfected controls (36).

These results make it imperative to measure vaccine effectiveness in PLHIV both to prevent damage to the health and wellbeing of PLHIV, and for effective control of the SARS-CoV-2 pandemic in South Africa.

In addition to HIV, older age and other prevalent comorbidities may affect vaccine effectiveness in the South African setting. It is well established that increasing age is associated with weakened immune responses and increased risk of infection, severe disease, and death. Obesity, hypertension, and diabetes mellitus are known risk factors for severe disease and death due to SARS-CoV-2 infection. In the Pfizer BNT162b2 vaccine phase II and III studies, just over 20% of people had comorbidities, most commonly hypertension, diabetes, and chronic lung disease. The vaccine showed similar efficacy in people with and without comorbidities and adverse events were not increased in those with comorbidities (37).

4.1.4. Homologous and Heterologous boost studies

Booster vaccination is required for multiple vaccines including most childhood vaccinations (38). With the exception of the J&J Ad26.CoV2 single dose vaccine, the majority of COVID-19 vaccines are comprised of a homologous two dose primary vaccine schedule, including the ChAdOx adeno-vectored and mRNA vaccines (39). Mouse models have demonstrated an increased immunogenic response with an adeno-vectored prime and mRNA vaccine boost compared to an adeno-vectored boost(40). Recently, compelling data has emerged that a heterologous boost with an mRNA-based vaccine such as the Pfizer BNT162b2 elicits a strong immunogenic response when following the adeno-vectored ChAdOx-1 vaccine prime (39, 41). In a recent phase II randomised trial in Spain, data on 676 participants who received a single ChAdOx-1 vaccine and were randomised to receive a Pfizer BNT162b2 boost was reported. The control group received no booster vaccine. The response ratio of heterologous boost:control arm was 77·69 (95% CI 59·57–101·32) for RBD protein and 36·41 (29·31–45·23) for trimeric spike protein IgG. In this study, participants reported mainly mild (68%) and moderate (30%) reactogenicity symptoms, most commonly injection site pain and induration, myalgia and headache with no serious adverse events reported (41). The CoM-COV study in the UK followed a prime-boost design with eight arms, including randomisation to ChAdOx-1/ ChAdOx-1, ChAdOx-1/Pfizer BNT162b2, Pfizer BNT162b2/ChAdOx-1 and Pfizer BNT162b2/ Pfizer BNT162b2 (39). In 463 participants, the ChAdOx-BNT162b2 regimen boosted anti-spike IgG 9.3-fold compared to those receiving two doses of ChAdOx-1 vaccine. Cellular responses, as measured by IFN-gamma ELISPOT, were also 3.9-fold greater in the heterologous prime-boost regimen (39). These results showed non-inferiority of the heterologous ChAdOx-1/Pfizer BNT162b2 prime-boost strategy, to the homologous ChAdOx-1/ChAdOx-1 strategy. In the Pfizer BNT162b2/ChAdOx-1 and Pfizer BNT162b2/ Pfizer BNT162b2 arms, heterologous boost did not achieve non-inferiority, but SARS-CoV-2 anti-spike IgG concentrations were higher in the heterologous arm (39).

The rate of systemic adverse events and paracetamol use in the two heterologous arms was higher than the homologous arms with 40 (36%) participants in the ChAdOx-1/ ChAdOx-1, 63/110 (57%) in the ChAdOx-1/Pfizer BNT162b2 arm, 48/117 (41%) in the Pfizer BNT162b2/ Pfizer BNT162b2 arm and 68/114 (60%) in the Pfizer BNT162b2/ChAdOx-1 reporting paracetamol use which mirrored reactogenicity (42). Safety blood tests showed similar results between the four arms (42). More recent data from the US, where 456 participants were enrolled who had received one of three covid-19 prime

vaccines (BNT162b2, J&J Ad26.COV2.S or mRNA-1273) received a homologous or heterologous booster vaccine at least 12 weeks after their prime vaccine. In this study, heterologous booster vaccines demonstrated an equivalent or higher binding and neutralising antibody response compared to heterologous, reactogenicity did not differ between the arms and there were no safety concerns(43). Additionally, prime-boost vaccination strategies with protein subunit adjuvant vaccines which include the SARS-CoV-2 spike protein subunit have been evaluated. In the CoM-Cov-2 study, people vaccinated in the community who had received either a BNT162b2 or ChAdOx-1 vaccine were randomly assigned to receive a booster with ChAdOx-1 or BNT162b2 or mRNA-1273 (Moderna) or NVX-CoV2373 (Novavax) vaccine. In this study GMC for anti-spike IgG was measured and non-inferiority demonstrated in the ChAdOx-1/Moderna and ChAdOx-1/Novavax arms compared to ChAdOx-1/ ChAdOx-1 arm. In the BNT162b2 prime group, BNT162b2/Moderna demonstrated non-inferiority but BNT162b2/Novovax did not. However, the GMC in the BNT/Novavax group was 18-fold increased compared to that of the ChAdOx-1/ ChAdOx-1 response at 28 days(44). Novavax can be transported and stored in less stringent conditions, requiring standard refrigeration which makes the vaccine particularly suitable for low-middle income countries (LMIC). A study evaluating a ChAdOx-1 prime with a MVC-COV1901 boost, also a protein subunit adjuvant-containing vaccine, demonstrated a significantly higher humoral and cellular response, compared to a homologous ChAdOx-1 booster (45). Additional data regarding the use of protein sub-unit vaccines is required, particularly in view of high background SARS-CoV-2 infections and previous heterologous boosters. In sum, heterologous prime-boost vaccination has the potential to be more cost effective since it increases vaccine efficacy, uses a lower cost prime, be more available since the adeno-vectored vaccines are more stable and available, be more easily stored if protein subunit vaccines are used and increase the effectiveness of vaccination. This approach warrants further evaluation particularly with the J&J Ad26.COV2.S vaccine as a prime, where data is not currently available. Bivalent vaccines which contain ancestral strain as well as omicron strain, such as the mRNA-1273.214 vaccine, used as a first or second booster in people who have completed their primary series of mRNA-1273 vaccine and a booster, elicit higher geometric mean titres against omicron strains, as well as other VOC with similar safety and reactogenicity profiles(46). Additional studies are underway. Although access to these vaccines is likely to take months yet, bivalent vaccines as a booster are exceptionally promising.

4.1.5. Fractional dosing of vaccines

Fractional dosing of vaccines has the advantage of reducing the amount of vaccine required. At an individual level, this has the potential to reduce reactogenicity and adverse events following immunisation. Reduced use of vaccines is advantageous in the context of vaccine shortages such as with COVID-19 vaccines and when vaccine prices remain high and unaffordable in many LMIC. The cost would be reduced, and the amount of available vaccines can be distributed more widely. This approach was used with the 17D yellow fever vaccine during the 2016 outbreak in Kinshasa where there was a shortage of yellow fever vaccine (47). One fifth fractional dosing of the vaccine was used for children over 2 years of age and non-pregnant adults. Fractional dosing had a good safety profile and at 1 month follow-up, 98% of participants overall were seropositive, and 98% of participants who were seronegative before receiving vaccine had seroconverted (47, 48). Seropositivity was sustained in 97% of the participants who remained in follow-up at 12 months (48). A similar approach is being evaluated for inactivated polio vaccine (IPV). A meta-analysis and systematic review by Mashunye et al., reported no significant difference in seroconversion when two or three doses of fractional IPV delivered intradermally, compared to full dose IPV given intramuscularly. Full dose IPV dose results in higher poliovirus type 1, 2 and 3 antibody titres, however the authors conclude that fractional IPV dosing is feasible and can reduce the demand on vaccine supply and potentially the associated cost (49).

Given the inequity in distribution and availability of COVID-19 vaccines, the associated cost and vaccine hesitancy, particularly in LICs where less than 10% of the population has been vaccinated, fractional

dosing particularly for a prime-boost strategy is advantageous. The Pfizer BNT162b2 vaccine has demonstrated a robust immunogenicity response, even at lower doses in phase 1 studies and since the adenovirus vectored-mRNA prime boost strategy has demonstrated good results with a ChAdOx prime and Pfizer BNT162b2 vaccine boost this strategy should be further explored (19, 41). The ongoing Phase 1/2a study (COV1001, NCT04436276) recently reported results after evaluating a full ($5x10^{10}$ viral particles [vp]) and quarter ($1.25x10^{10}$ vp) J&J Ad26.COV2.S boost at 6 months post Ad26.COV2.S prime (15). The full dose booster elicited a 9-fold increase in spike binding protein antibodies at 7 days post boost compared to 29 days post prime, and the quarter dose a 6-7.7 fold increase at day 28 post boost, compared to day 29 post prime (15). The response to quarter dose boost was slower in participants over 65 years, but at 28 days was similar in younger and older participants (15). Solicited local adverse events were similar between the prime and quarter dose booster, while solicited systemic adverse events were lower after the boost, 61.7% vs 28% (15).

The BaSiS primary study evaluated a prime-boost strategy in HCW and people vaccinated through the SA vaccination programme in South Africa, who received a J&J Ad26.COV2.S vaccine prime through the SISONKE phase IIIB implementation study and routine programmes, receiving either a full dose Ad26.COV2.S booster ($5x 10^{10}$ vp/ml) or a half dose Ad26.COV2.S booster ($2.6x 10^{10}$ vp/ml) or a full (30mcg) or half (15mcg) dose Pfizer BNT162b2 booster vaccine. The study includes people over 18 years of age, regardless of HIV status. The extension study builds on formative work in the primary study, extending visits through 24 months, focussing on PLHIV, a population of key importance for the South African vaccination programme.

4.2. Rationale for the primary study:

1. Ad26.COV2.S prime alone triggers lower titres of neutralising antibodies than mRNA vaccines.
2. Other heterologous combinations of adenovirus vectored prime (ChAdOx) and RNA vaccine boosts (Pfizer BNT162b2) are highly immunogenic.
3. Although data exists for J&J Ad26.COV2.S vaccine prime followed by an mRNA vaccine boost, data including fractional homologous and heterologous dosing remains scarce.
4. There is a scarcity of data regarding the immunogenicity of vaccines in PLHIV, who account for a significant proportion of South Africans.
5. People over the age of 55 years have a progressively weakened immune response to vaccines and may benefit from a homologous or a heterologous prime boost.
6. People living with HIV have compromised immune responses even if well controlled on ARVs and may benefit from a homologous or heterologous prime boost.
7. There is public health benefit, including cost and increased availability for greater numbers of people, in addition to potential reduction in local and systemic adverse effects, with fractional dosing for booster vaccines. Available data, although limited, demonstrates that fractional dosing results in robust immune responses.

4.3. Objectives and endpoint measures for BaSiS Primary study

For the primary study, the following objectives were included, and these analyses are underway, tabulated in table 3:

Primary Objectives

- To evaluate the immunogenicity of a homologous vaccine boost with either a full ($5x 10^{10}$ vp/ml, 0.25 ml) or a half dose ($2.6x 10^{10}$ vp/ml, 0.13 ml) J&J Ad26.COV2.S, or a heterologous boost, with either a full dose (30mcg, 0.3ml) or a half dose (15mcg, 0.15 ml) Pfizer

- BNT162b2 vaccine, following J&J Ad26.COV2.S vaccine administered through the SISONKE phase IIIB implementation study or South African Covid-19 vaccination programme, by comparing antibody and T cell responses before and after boosting.
- To evaluate safety and reactogenicity after a half or full dose J&J Ad26.COV2.S or Pfizer BNT162b2 vaccine booster dose.

Secondary objectives

- To assess whether length of time between prime and booster dose impacts immunogenicity.
- To assess differences in immunogenicity by age and by HIV status.
- To evaluate boosted antibody responses against ancestral and novel SARS-CoV-2 strains including D614G, beta, delta, and other variants of concern (VOC) compared to baseline.
- To evaluate the capacity of boosted T cell responses against ancestral and novel SARS-CoV-2 strains including D614G, beta, delta, and other relevant VOC as they emerge compared to baseline.

Exploratory objective

- To evaluate whether clotting profiles in participants at baseline and 2 weeks differ by booster arm, HIV status and age.

Table 3: BaSiS Primary Study Objectives and endpoint measures

	Objectives	Endpoint measures
Primary	Immunogenicity (antibody and T cell)	Neutralisation titres post-boost versus baseline using the pseudovirus neutralisation and live virus neutralisation assays. T cell response magnitudes post-boost versus baseline using intracellular cytokine staining.
	Safety and reactogenicity	Local and systemic reactogenicity measured by diary cards.<br>Monitor ARs, SAEs, SUSARS, AESI
Secondary	Evaluate impact of duration from prime to boost on immunogenicity	Antibody and T cell immunogenicity in each group from time of prime to boost
	Differences in immunogenicity by HIV status and age	Comparison of antibody and T-cell response between PLHIV and HIV-uninfected and those ≥ 55 years and < 55 years
	Evaluate cross-reactivity of boosted neutralising antibody responses to ancestral and novel SARS-CoV-2 variants compared to baseline	Pseudovirus and live virus neutralising antibody titres pre and post booster vaccine, tested against D614G, delta, beta (and other VOC should these become locally relevant)
	Evaluate cross-reactivity of T cell responses to ancestral and novel SARS-CoV-2 variants compared to baseline	T cell responses to D614G, delta, beta (and other VOC should these become locally relevant)
Exploratory	Evaluate differences in clotting profiles with different boosters, age and HIV status	Compare differences between haemoglobin, platelets and D-dimer at baseline and 2 weeks in each study arm and according to HIV status and age

4.4. Progress on BaSiS Primary Study

The study arms and numbers of participants enrolled in each have been described in Table 4.

This design has provided data to determine if a boost of J&J Ad26.COV2.S or Pfizer BNT162b2 mRNA vaccine triggers high levels of nAbs and T cells, and determine whether the J&J Ad26.COV2.S and Pfizer BNT162b2 dosage can be reduced for cost effectiveness/vaccine sparing, and reduction in local and systemic adverse events post-vaccination.

Immunogenicity was quantified by measuring nucleocapsid binding antibodies (to assess prior infection) and neutralisation capacity of blood plasma, a measure highly correlated to vaccine efficacy (16). Trial participants had blood draws at baseline drawn immediately before vaccination, and then followed up at 2 weeks, 3 months and 6 months post vaccination to test peak immunogenicity and durability. Peripheral blood mononuclear cells will be isolated for evaluating T cell immune responses.

Binding antibodies were determined by ELISA to the nucleocapsid. Neutralisation capacity were determined independently by the laboratory of Penny Moore at NICD using a pseudovirus neutralisation assay (PNA) and Alex Sigal at Africa Health Research Institute using a live virus neutralisation assay (LVNA) by established protocols (50). SARS-CoV-2 variants to be neutralised included the ancestral reference strain and the beta, delta and omicron variants for all samples, as well as other variants which may emerge for select samples. Assays will be completed once the effect of boost on neutralisation at the tested significance level outlined in the analysis plan is determined. T cell responses to the ancestral, beta, delta, omicron and other VOC are being measured by Wendy Burgers at the University of Cape Town, as described (51). Interim results are being analysed for the 2 week, 3 months post-boost timepoints as well as the full analysis through 6 months to assess durability of the booster immune response.

Trial sites included the Wits RHI Shandukani and PHRU Kliptown sites in Johannesburg, the CAPRISA eThekwini Clinical Research Site in Durban and the Desmond Tutu Health Foundation site in Masiphumelele in Cape Town.

Table 4: Distribution of participants enrolled in the BaSiS study by group and site

Site	Group A	Group B	Group C	Group D	**TOTAL**
Wits RHI	26	25	26	25	**102**
CAPRISA	6	6	8	7	**27**
PHRU	15	13	14	14	**56**
Masiphumelele	27	25	25	27	**104**
**TOTAL**	**74**	**69**	**73**	**73**	**289**

In table 5 we describe the baseline characteristics of participants enrolled on the BaSiS study. The majority of participants were 30-54 years of age, however 12.5% were ≥ 55 years of age. Over 40% of participants were PLHIV, 18% considered to be poorly controlled based on CD4 count < 350 cells/mm^3 or elevated VL. In the study 9 participants had breakthrough infections.

Table 5: Baseline demographic and clinical characteristics of participants enrolled in BaSiS

	J&J Ad26.COV2.S Full dose		J&J Ad26.COV2.S Half dose		Pfizer BNT162b2 Full dose		Pfizer BNT162b2 Half dose		Total	
	n	%N	n	%N	n	%N	n	%N	n	%N
**Enrolled, randomised and vaccinated (N)**	74	25.6%	69	23.9%	73	25.3%	73	25.3%	289	100.0%
**Age groups**										
**18-29 years**	5	6.8%	5	7.2%	7	9.6%	6	8.2%	23	8.0%
**30-44 years**	41	55.4%	46	66.7%	34	46.6%	34	46.6%	155	53.6%
**45-54 years**	20	27.0%	10	14.5%	23	31.5%	22	30.1%	75	26.0%
**55+ years**	8	10.8%	8	11.6%	9	12.3%	11	15.1%	36	12.5%
**Sex**										
**Males**	10	13.5%	8	11.6%	20	27.4%	15	20.5%	53	18.3%
**Females**	64	86.5%	61	88.4%	53	72.6%	58	79.5%	236	81.7%
**Ethnicity**										
**Black African**	67		66		69		68		270	93.4%
**Other**	5		1		3		2		11	3.8%
**Unknown**	2		2		1		3		8	2.8%
**HIV status at enrolment**										
**HIV Uninfected**	47	63.5%	41	59.4%	42	57.5%	43	58.9%	173	59.9%
**PLHIV - known**	27	36.5%	27	39.1%	28	38.4%	30	41.1%	112	38.8%
**PLHIV - new**	0	0.0%	1	1.4%	3	4.1%	0	0.0%	4	1.4%
**VL elevation (>1000 copies/ml) among known PLHIV**										
**Suppressed**	27	100.0%	23	85.2%	27	96.4%	24	80.0%	101	90.2%
**Elevated**	0	0.0%	4	14.8%	1	3.6%	4	5.5%	9	8.0%
**Missing***	0	0.0%	0	0.0%	0	0.0%	2	2.7%	2	1.8%
**VL elevation (>40 copies/ml) among known PLHIV**										0.0%
**Suppressed**	26	96.3%	23	85.2%	23	82.1%	24	80.0%	96	85.7%
**Elevated**	1	3.7%	4	14.8%	4	14.3%	5	6.8%	14	12.5%
**Missing***	0	0.0%	0	0.0%	2	7.1%	0	0.0%	2	1.8%
**CD4 count among known PLHIV (cells/mm3)**										
**>=350**	24	88.9%	22	81.5%	25	89.3%	24	80.0%	95	84.8%
**<350**	2	7.4%	4	14.8%	2	7.1%	4	5.5%	12	10.7%
**Missing****	1	3.7%	1	3.7%	1	3.6%	2	2.7%	5	4.5%
**HIV disease not well controlled*****	2	7.4%	6	22.2%	6	21.4%	6	20.0%	20	17.9%
**On ART, among known PLHIV**	27	100.0%	26	96.3%	28	100.0%	29	96.7%	110	98.2%
**Median time between Prime and Booster**	264.5	(245-292)	271	(259-302)	273	(258-290)	269	(252-294)	270	(254-293)
**Visits Completed**										
**2 Week**	70	94.6%	65	94.2%	70	95.9%	69	94.5%	274	94.8%
**3 Month**	57	77.0%	55	79.7%	55	75.3%	56	76.7%	223	77.2%
**2 Week & 3 Month**	57	77.0%	55	79.7%	55	75.3%	56	76.7%	223	77.2%
**6 Month**	28	37.8%	28	40.6%	27	37.0%	28	38.4%	111	38.4%

Footnote: * VL missing - 2 new enrolments
** CD4 missing - 4 new enrolments, 3 newly diagnosed, 1 known positive but did not disclose to investigator, so treated as new diagnosis and no CD4 conducted
***VL >40 copies/ml or CD4<350 cells/mm3

4.5. BaSiS Primary study Interim results

A DSMB for the study to evaluate safety and early immunogenicity was held on the 24th August 2022. Early interim results from the 2-week and 3-month visits showed no safety concerns for any of the regimens, with 53.6% of people overall reporting solicited reactogenicity adverse events, 47.4% local and 41.2% systemic, with slightly higher events in the Pfizer arms. There were 9 documented breakthrough infections on study, all mild, 7 in the J&J arms. Although only 30.8% of people reported previous SARS-CoV-2 infection, 90% of participants had positive nucleocapsid antibodies at enrollment, highly reflective of hybrid immunity. The spike binding antibody titers at 2-weeks and 3-months were higher for those boosted with Pfizer vaccines, with no difference between HIV-negative participants and those living with HIV. A similar trend was seen with neutralizing antibodies, where cross reactivity across variants was demonstrated. Using live virus neutralization assays Pfizer full dose had a higher-fold response compared to the other arms, and again no significant difference in PLHIV. Similarly, highest spike-specific CD4+ T cell responses were seen in the full dose Pfizer group at 2-weeks with no difference in PLHIV. In the CD8 response, boosting only occurred in the HIV-negative group who received a full dose Pfizer boost. Based on these data, the DSMB adjudicated that a Pfizer full dose vaccine booster should be offered to all participants except those who received a full dose Pfizer vaccine <6 months prior. All the relevant HRECs have reviewed the DSMB reports and approved the submitted "Dear Participant" letter and the booster vaccinations will be offered to participants and completed by 31st January 2023. Current Pfizer vaccine available in South Africa expires 31 January 2023. Participants will receive a diary card to collect safety data, but no additional blood draws post-booster are planned until the 12 month-visit as part of the extension study detailed below. In addition, further analyses to describe non-responders as well as to evaluate the responses in a larger number of participants at the 2-week, 3-month and 6-month time points are underway.

5. BaSiS Extension study

In the BaSiS extension study, we build upon the pre-existing work from the primary study, extending study visits through 12, 18 and 24 months and conducting additional laboratory testing in the cohort. The BaSiS Extension objectives and endpoints are tabulated below in Table 6.

In the extension study, based on available data regarding VOC, vaccine availability, and participant data we will explore providing an additional booster vaccine which in extension follow-up (extension booster). Given currently available data, potential vaccines will include the Covovax vaccine, an additional Pfizer BNT162b2 vaccine or a bivalent vaccine if available (Table 7).

Table 6: Objectives and endpoint measures for the BaSiS Extension study

Objectives	Endpoints
1. Long term follow-up of the BaSIS cohort including additional visits at **12 months, 18 months and 24 months compared to baseline.**	Determine immunogenicity for each vaccine prime/boost combination by evaluating neutralisation titres post-boost versus baseline
2. Monitor long-term humoral immunity in PLHIV after vaccine boosting	Immunogenicity for each vaccine prime/boost combination in PLHIV

3. Characterize SARS-CoV-2 viruses in breakthrough infections	Viral sequence/variant contribution to BTI in each prime/boost regimen
4. Monitor long term immunological memory in PLHIV	Determine whether PLHIV have inferior long term T cell memory responses compared to HIV-uninfected people
5. Perform HLA typing of the cohort to understand T cell breadth, specificity and escape potential	Characterize breadth, specificity and cross-reactivity of the T cell response after vaccine boosting

5.1. Rationale for extension of study to 12, 18 and 24 month visits:

5.1.1. Long term follow-up of BaSiS participants

- Although data are available on short term immune (<6 months) responses to prime/boost vaccination, data on long term durability are not readily available in the South African population and is particularly limited in PLHIV. PLHIV may benefit from an optimized vaccine regimen including additional heterologous or homologous vaccines.

- It is important to determine the immune response in the first year post full dose Pfizer booster vaccine and to evaluate long-term memory response.

- If the majority of participants receive a full dose Pfizer vaccine in the next few months per version 2.0, to conclude by 31 January 2023 in response to interim data and DSMB recommendation as planned, immunogenicity and durability can be evaluated over a longer follow-up period. Long term immunogenicity and durability will be particularly important in PLHIV, 40% of the cohort. The current blood draw schedule is built around additional visits at 12, 18 and 24 months providing additional time points to assess durability.

- It will be important to understand how immunity to the virus develops as a combination of boosting and natural infection which is likely to occur in the study population.

- Given the likelihood of emergence of new VOC, the study will allow testing of immune responses against emerging variants.

- Long term effects of comorbidities such as HIV, TB and diabetes on immune memory and durability can be tracked through long-term follow up.

- It is important to establish longer-term adverse reactions with a J&J Ad26.COV2.S-Pfizer BNT162b2 prime-boost strategy, given that this information cannot be extrapolated from current clinical trials. Such information will assist with decision-making regarding prime-boost strategies in South African and sub-Saharan African countries where this strategy may be most likely given the currently available vaccines being offered in the rollout.

- The extension strategy was recommended by the BaSiS study DSMB.

5.1.2. Rationale for HLA typing of all participants

HLA typing will complement the T cell immunogenicity studies being performed in BaSIS, enabling HLA associations with CD8 T cell variant escape, hyporesponsiveness and epitope identification efforts (described below).

5.1.3. Rationale for isolating and characterizing the infecting virus from breakthrough infections (BTI)

In the extension study, nasopharyngeal swabs will be collected at each study visit to identify any asymptomatic breakthrough infections. These samples will be sent to the Sigal lab for sequencing and isolation of the infecting virus. The isolated viruses will be examined for immune escape. This component will address virus specific reasons for BTI, including any changes to viral sequence/ infecting variant and should enable us to understand whether relative immunogenicity of a given vaccine regimen relative to the others tested is virus/variant specific.

BaSiS study objectives and endpoint measures are listed in **Table 6** above

5.2. Approach per extension study objective

5.2.1. Objective 1. Long term follow-up of the BaSiS cohort

Outcome 1: Determine immunogenicity for each vaccine prime/boost combination

We will establish whether a heterologous or a homologous prime/boost combination results in improved immunogenicity as measured by antibody neutralization capacity. Furthermore, we will determine whether a half-dose homologous or heterologous boost gives similar immunity to a full dose in terms of peak antibody neutralization response and durability of neutralization. **Table 7** outlines the schedule of evaluations, with Extension study visits **bolded.** Immunogenicity (binding, neutralization and functional antibody and T cells) will be performed at the additional visits. We will explore where anti-vector Ad26 antibodies and T cells have an impact on lower early humoral and cellular immune responses described in the interim analysis.

Given the likelihood of continued viral evolution and the emergence of variants and subvariants of unknown properties, the 24 months of follow-up in the BaSiS Extension study will afford us the opportunity to examine immunologically the complex exposure histories that will arise over the 24 months of participant follow-up. For antibodies, this is of particular relevance given the emerging evidence of affinity maturation post-vaccination (and after infection) which drives enhanced breadth of plasma responses. Measuring specific antibody and T cell responses in the same individuals will provide a powerful measure of the co-ordinated immune response in the context of hybrid immunity. To date, 9 BTI have been identified in the BaSiS cohort. BTI, along with clinical outcome data, will afford us the opportunity to track immune responses with reference to immune protection and escape.

Table 7: Revised schedule of evaluations in BaSIS

	Screening/ enrolment (Day 0)	2 weeks	3 months	6 months	**12 months**	**18 months**	**24 months**	**Interim visit [a]**
Visit window	NA	±3 days	±7 days	±14 days	**± 28 days**	**± 28 days**	**± 28 days**	N/A
ICF or ICF Amendment	X			**X[b]**	**X[b]**			

Weight, height, body mass index	X				**X**			
Vital signs [c]	X	X	X	X	**X**	**X**	**X**	X
Medical history	X	X	X	X	**X**	**X**	**X**	X
COVID-19 infection related history	X	X	X	X	**X**	**X**	**X**	X
Targeted physical examination if necessary	X	X	X	X	**X**	**X**	**X**	X
Contraception history, LMP and Urine BHCG in WOCP	X				**X**[d]	**X**[d]	**X**[d]	
Eligibility check	X			X[e]	**X**[e]			
Vaccination check[f]	X	X	X	X	**X**	**X**	**X**	X
HIV Elisa test [g]	X				**X**		**X**	
FBC and D-Dimers	X	X						
Immunology bloods in PLHIV (CD4, CD8, FBC, lymphocytes, neutrophils) [h]	X			X	**X**	**X**	**X**	
HIV-1 Viral Load in PLHIV[h]	X	X		X	**X**	**X**	**X**	X
Immunogenicity bloods-SARS-CoV-2 antibodies [i]	X	X	X	X	**X**	**X**	**X**	X
Immunogenicity bloods-Cellular immunity (PBMC) [i]	X	X	X	X	**X**	**X**	**X**	
Nasal swabs SARS-CoV-2 PCR	X				**X**	**X**	**X**	X
HLA Testing					**X**[j]	**X**[j]	**X**[j]	
Randomization	X							
Vaccination	X				**X**[k]	**X**[k]	**X**[k]	
Post-vaccination observations (15+ mins)	X				**X** [k]	**X**[k]	**X**[k]	
Train on and issue vaccine diary card	X							
Train on & issue memory aid	X							
Diary card collection		X	X					
Collect solicited and unsolicited AEs		X	X	X	**X**[l]	**X**[l]	**X**[l]	X[l]
Blood volumes	PLHIV: 70mL HIV-: 60 mL	60 mL (+ 6 mL for newly diagnosed PLHIV)	50 mL	PLHIV: 70 mL HIV-: 60 mL	**70 mL**	**70 mL**	**70 mL**	70 mL

Footnote:

[a] Interim visits to include:

- *Unsolicited AEs throughout the study post vaccination < day 28 or if they are grade 3 or higher or SAEs throughout study*
- *Solicited reactogenicity ongoing at day-7 post vaccination, or unsolicited if related day-7 post vaccination*
- *Participant has any symptoms suggestive of SARS-CoV-2 infection or tests positive for SARS-CoV-2 external to the study*

[b] *Informed Consent amendment signed at the 6 month visit (for participants still on the main study) or 12 month visit (for participants already off the main study). Includes Main ICF, Storage ICF, HLA testing ICF and HIV testing ICF for people not known to be PLHIV.*

[c] *Vital signs conducted: Temperature, blood pressure, respiratory rate, heart rate, oxygen saturation*

[d] *On vaccination day for extension booster in WOCP*

[e] *Brief eligibility check at first visit after extension ICF signed.*

[f] *Vaccination check (COVID-19 vaccine and non-Covid-19 vaccine) will be conducted at each extension visit and documented in the conmed log. For the extension study, addition vaccinations external to study are allowable and will be clearly documented and recorded in the database.*

[g] *Counselling and referral for HIV care including antiretroviral therapy if a participant tests positive on HIV Elisa.*

[h]VL, CD4 and additional bloods will be done in people who are diagnosed with HIV at the 12-month or 24-month visit on study, or if diagnosed external to the study.
[i]Elisa testing will be done on all samples, neutralisation assays will only be done on participants who test positive on Elisa. PBMC samples will be taken for all participants to test cellular immune responses.
[j] HLA testing will be conducted on stored samples once informed consent completed as part of the extension study
[k] An additional booster vaccine (Extension booster) will be offered at one of the extension visits for participants who were on either half dose arm or full dose Ad26.CoV2.S arm, as described in this protocol.
[l]AEs: Participants will be requested to notify the study team if any solicited reactogenicity responses continue beyond day 7 post-vaccination, any unsolicited AEs occur within 28 days and for any SAEs throughout study follow up.

5.2.2. Objective 2. Monitor long-term humoral immunity in PLHIV after vaccine boosting

Outcome 2: Immunogenicity for each vaccine prime/boost combination in PLHIV

We will determine which prime/boost combination has the highest immunogenicity in PLHIV and whether fractional doses elicit similar immunity to full doses in this group both in peak antibody neutralization response and in the durability of the neutralization.

Approach: a live virus neutralization assay (LVNA) is required for this objective to avoid HIV antiretroviral therapy inhibition of the pseudoviral vector backbone used in this study. This assay will measure the number of infection foci formed upon SARS-CoV-2 infection of an H1299 cell line engineered to express the ACE2 receptor (H1299-ACE2). Vero cells are not used as they do not infect well with live Omicron sub-lineages. Viruses tested will be ancestral SARS-CoV-2 with the D614G substitution and the Omicron BA.5 sub-lineage. In the event where another variant becomes dominant and BA.5 becomes extinct, we will substitute the dominant variant for BA.5. The LVNA involves the addition of virus at approximately 100 focus forming units per well to cells at confluence in a 96-well format plate. Before addition of the virus to the cells, it is incubated with 8 1:2 serial dilutions of plasma from each study participant. Dilutions start at 1:25 (most concentrated) but can start at 1:10 if neutralization capacity is weak, or 1:250 if neutralization capacity is very strong. The reduction in foci relative to the now plasma control is then fitted to a sigmoidal curve to obtain the reciprocal of the plasma dilution giving 50% inhibition ($FRNT_{50}$). The live virus neutralization assay will be compared to Elisa testing in the same cohort.

Our preliminary results investigating the effect of vaccination in PLHIV show that response to the Pfizer BNT162b2 vaccine may be dependent on whether HIV viremia is suppressed (**Figure 1**). Data from the proposed study would enable us to quantify the effect of HIV status and suppression on neutralizing immunity elicited by the different prime/boost combinations and help determine which of the combinations is best optimized for PLHIV.

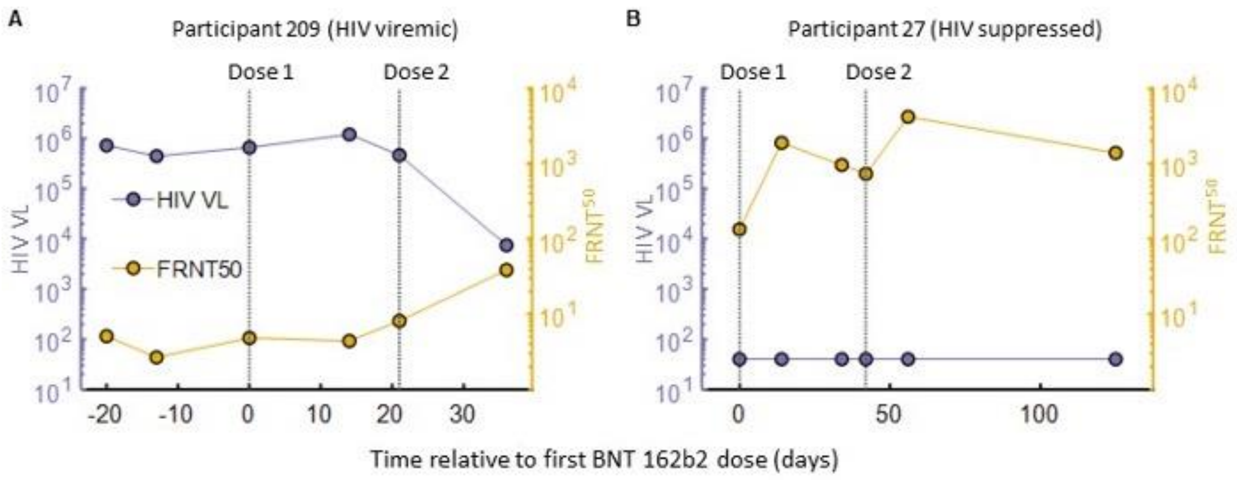

Figure 1: Example of response of two people living with HIV to Pfizer BNT162b2 vaccination. **(A)** HIV viral load (VL, RNA copies/mL) and neutralization capacity as $FRNT_{50}$ against ancestral/D614G virus for participant 209 enrolled in the AHRI cohort who had high HIV viremia. **(B)** HIV VL and $FRNT_{50}$ against ancestral/D614G virus for participant 27 enrolled in the AHRI cohort who had suppressed HIV viremia. Neutralization as $FRNT_{50}$ determined using a live virus assay. Both participants had low CD4 counts (<200 cells/uL) and were previously infected with SARS-CoV-2. Left y-axis is HIV VL, right y-axis is neutralization capacity as $FRNT_{50}$. Timing of vaccine doses is denoted by dashed vertical lines.

5.2.3. Objective 3. Characterize SARS-CoV-2 viruses in breakthrough infections

Outcome 3: Viral sequence/variant contribution to BTI in each prime/boost regimen

BTI may occur due to virus specific factors such as emerging mutations or variants, and different vaccine regimens may elicit different breadth of infection - the degree to which cross-protective responses are elicited.

To examine the properties of SARS-CoV-2 breaking through virus, we propose to sequence and isolate live BTI viruses and test them against panels of plasma samples from vaccinated, variant infected, or infected/vaccinated participants. Each panel consists of plasma samples from 20-30 study participants vaccinated with BNT162b2, vaccinated with BNT162b2 with previous infection of ancestral/D614G virus, vaccinated and infected with BA.1, BA.2, or BA.5, and infected with BA.1, BA.2, and BA.5 only. These data will be important in characterizing emerging BTI variants and give insight into whether different prime/boost combinations have a different effectiveness against emerging variants.

Approach: We will obtain nasopharyngeal swabs from participants with breakthrough infections and, after filtering, add the viral transport medium from the swabs to a culture of H1299-ACE2 cells. After incubating for four days (passage 1, P1), infected cells will be detached and added to a culture of Vero E6 cells. An additional 4-day incubation will complete passage 2 (P2) and virus will be used from this stage for LVNA using the plasma panels to determine viral determinants of immune escape of breakthrough virus (see Objective 2 for methodology).

A previous collaboration between the Sigal and Moore labs showed the insight gained from a combined serology, viral isolation, and sequencing approach in a longitudinal study design (**Figure 2**).

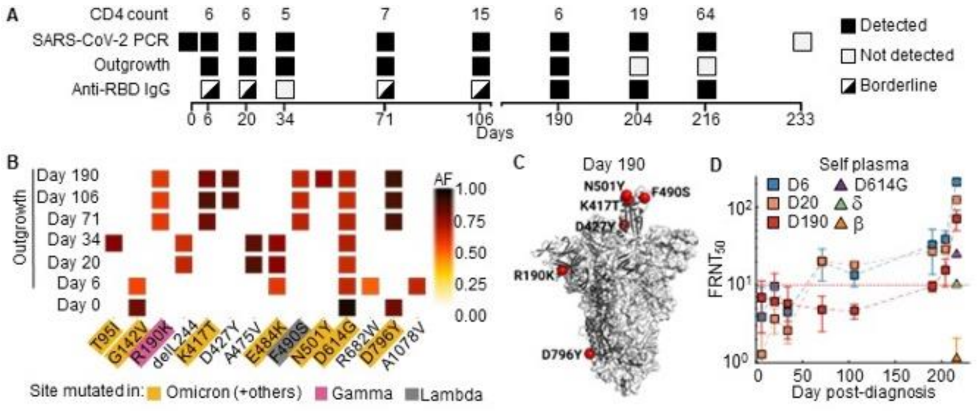

Figure 2: Mapping neutralization of evolved virus in an individual with advanced HIV disease. (A) Participant characteristics over 233 days from SARS-CoV-2 infection: CD4 T cell count (cells/µL), SARS-CoV-2 by qPCR, virus outgrowth, and presence of anti-RBD IgG. **(B)** SARS-CoV-2 genotypes in the swab (day 0) and outgrowth (day 6 - 190). AF: allele frequency. **(C)** Cryo-EM structure of the SARS-CoV-2 spike protein. Mutations in day 190 isolated virus are shown as red spheres**. (D)** Neutralization of day 6 isolated, day 20 isolated, and day 190 isolated virus by self-plasma collected days 6 to 216 and ancestral D614G, Beta and Delta viruses with plasma from day 216.

5.2.4. Objective 4. Monitor long term immunological memory in PLHIV

Outcome 4: Determine whether PLHIV have inferior long term T cell memory responses compared to HIV-uninfected people

There is evidence that some vaccines elicit suboptimal responses in PLHIV. While several studies describe antibody responses in this population, fewer have characterized T cell immunity or the durability of cellular memory in PLHIV following vaccination for COVID-19. Despite viral suppression by ART, incomplete CD4 T cell reconstitution, residual immune activation and persistent T cell exhaustion may compromise immune memory in PLHIV. Our general understanding is that T cell responses provide protection against severe outcomes of COVID-19, particularly in the face of emerging variants that effectively escape the neutralizing antibody response. Inadequate T cell responses or accelerated waning of vaccine T cell responses may thus increase risk of severe disease over time. Understanding the durability of T cell memory in PLHIV is thus critical for guiding vaccination strategies.

We have examined T cell responses in the Sisonke substudy, comparing PLHIV (n=75, all on ART) to HIV-uninfected participants (n=168), 6 weeks after a single dose of Ad26.CoV2.S. Using intracellular cytokine staining and flow cytometry, we did not detect any defect in the magnitude of CD4 and CD8 IFN-g T cell responses at this early time point after vaccination either in those who had prior COVID-19 or not (**Figure 3**). In the naïve vaccinees (without a spike response at baseline), however, there were significantly fewer PLHIV who mounted a CD8 T cell response (49% vs. 69%, p=0.014), which may be consistent with inferior CD4 T cell help for inducing CD8 responses in some individuals. Further detailed analysis, including the 24 week post-vaccination time point, is underway. It will be important to determine whether defect is present in those who have had two vaccine doses in BaSiS.

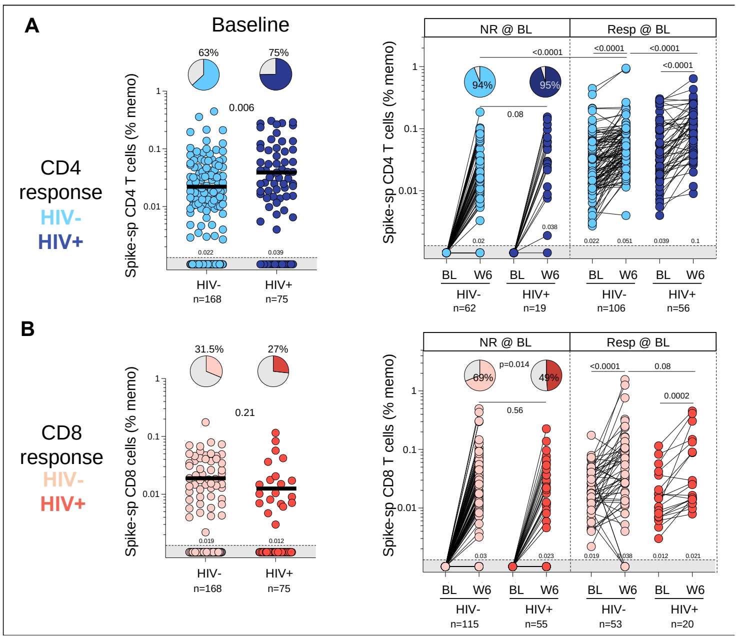

Figure 3: Comparison of SARS-CoV-2 spike-specific T cell responses in PLHIV and HIV-uninfected participants in the Sisonke substudy (n=243). Frequencies of SARS-CoV-2 spike-specific CD4 (blue) **(A)** and CD8 (red) **(B)** T cell responses (cells producing IFN-ɣ), measured 6 weeks after single dose of Ad26.CoV2.S. The left panel shows responses at baseline, with positive responses indicating prior COVID-19. The right panel shows vaccine responses in those without prior COVID (NR), as well as those with a detectable response at baseline (Resp). Horizontal lines indicate median frequencies of responders (values are indicated on the graphs) and pie charts indicate the frequency of responders. NR: Non-responders at baseline. Resp: Responders at baseline (detectable spike T cell response pre-vaccination).

We will characterize T cell responses at baseline, 2 and 24 weeks after Ad26.CoV2.S/BNT162b2 boost. The BaSiS Extension will enable us to additionally monitor T cell immunity in all participants (extra n=100, total n=300), as well as extend immune measurement to 12 and 24 months after vaccination in the whole cohort. Increasing the number of participants with T cell measurements increases the power of subgroup analyses. To identify potential defects in immunity in PLHIV, we will examine both quantitative features of spike-specific T cells as well as qualitative characteristics, including polyfunctional ability, cytotoxic potential and memory differentiation profiles (proportions of long-lived and short-lived memory subsets), using multiparameter flow cytometry.

5.2.5. Objective 5. Perform HLA typing of the cohort to understand T cell breadth, specificity and escape potential

Outcome 5: Characterize breadth, specificity and cross-reactivity of the T cell response after vaccine boosting

Human leukocyte antigens (HLA) are central to the immune responses to infectious diseases, determining the peptide repertoire presented to T cells in an individual. African HLA alleles are highly diverse, with novel alleles and differences in allele frequency and distribution compared to other ethnicities. Epitopes targeted in Africans and their HLA restrictions are under-represented in epitope discovery databases, limiting the use of epitope-HLA prediction tools. Thus, SARS-CoV-2 epitope identification is important to advance vaccine science. The BaSiS Extension study includes storage of blood pellets at the 6 month visit on some but not all participants, from which we will perform genomic DNA extraction and high-resolution HLA Class I and II typing using Illumina Miseq. For those who do not have samples stored from the 6-month visit, blood pellets will be stored from a sample taken at either the month 12, 18 or 24 visit.

Our preliminary data demonstrate that while most individuals mount a T cell response to spike after repeated exposures from infection or vaccination **(Figure 4)**, the magnitude of the spike-specific CD4 and CD8 T cell response is unchanged, in contrast to antibody responses. The lack of increase in magnitude would suggest that the breadth of the response does not increase with multiple spike exposures but is rather boosted to original levels (an immunological 'ceiling') after waning. However, further studies are warranted in longitudinal cohorts. Understanding the breadth of the response after vaccination, and how it is shaped by prior or subsequent (breakthrough) infection, is important for providing insights into cross-reactivity to variants and the likelihood of T cell escape: a dominant response targeting a few spike epitopes may be more susceptible to viral escape due to mutational change in those few epitopes, compared to a response targeting a larger number of epitopes, or one that broadens over time with each exposure. Only a handful of studies have examined breadth after vaccination and notably, an advantage of heterologous vaccine boosting over homologous boosting has been observed. No studies have addressed breadth of response in PLHIV.

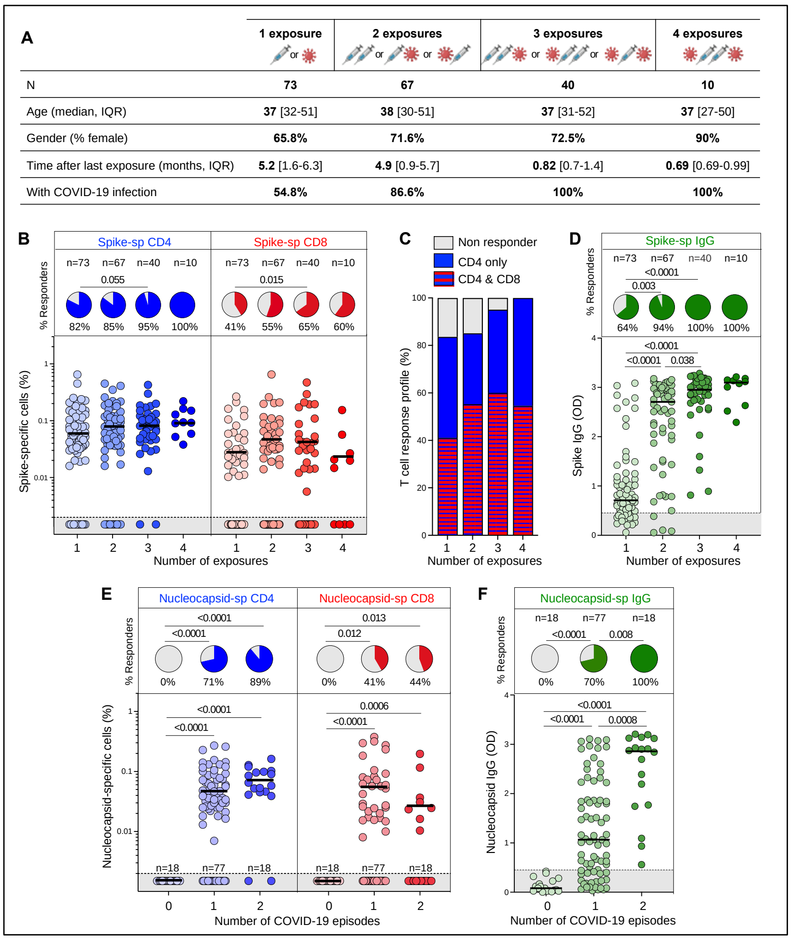

Figure 4: Comparison of SARS-CoV-2-specific T cell and IgG responses upon repeated exposures to SARS-CoV-2 antigens. (A) Clinical characteristics of participants grouped according to their number of

exposures. The syringe corresponds to Ad26.CoV.S vaccination and the virus depicts a COVID-19 episode. **(B)** Frequencies of ancestral SARS-CoV-2 spike-specific CD4 (blue) and CD8 (red) T cell responses (i.e., cells producing IFN-γ, TNF-α or IL-2) in individuals with an increasing number of exposures. Horizontal lines indicate median values of responders. **(C)** Profile of the ancestral spike-specific T cell response based on SARS-CoV-2 antigen exposures. **(D)** SARS-CoV-2 spike-specific IgG measured by ELISA. **(E)** Frequencies of the SARS-CoV-2 nucleocapsid-specific CD4 and CD8 T cell responses. **(F)** SARS-CoV-2 nucleocapsid-specific IgG. Pies depict the proportion of participants exhibiting a detectable T cell response. Statistical analysis was conducted using the Kruskal-Wallis with Dunn's multiple comparison test between the different exposures.

We will investigate whether antigenic imprinting occurs (primarily a boosting of initial response specificities at baseline) or whether *de novo* T cell responses are generated after vaccine boosting by mapping individual reactive spike peptides in a subset of BaSiS participants (n=80 at baseline and post-vaccination). Using established methods, we will perform peptide pulsing and 10 day *in vitro* culture (**Figure 5**), to obtain sufficient cells for single peptide mapping and confirmation. We have obtained successful expansion of spike-specific cells (170 to 300-fold expansion), demonstrating the feasibility of this approach. We will map individual reactive spike peptides in each individual.

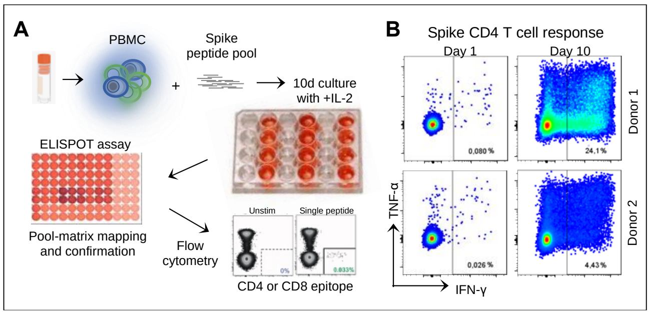

Figure 5: Mapping approach to identify single reactive spike peptides**. (A)** Thawed PBMC are stimulated with peptides (e.g., 253 overlapping peptides covering the full spike, or variant pools), washed, cultured for 10 days in the presence of IL-2 and harvested on day 10. Single reactive peptides are identified and confirmed using an IFN-g ELISPOT assay, and recognition by CD4 or CD8 T cells determined by flow cytometry. **(B)** Representative plots showing IFN-g and TNF-a CD4 T cell frequencies on Day 1 (non-expanded) and Day 10, demonstrating 170 to 300-fold expansion of the spike T cell response.

A complementary approach to determine broadening of the response is through T-cell receptor (TCR) sequencing. We have not included TCR sequencing in this proposal, since we do not have it established in our laboratory, and are currently evaluating what might be the best approach to determine TCR repertoires before and after vaccination (or BTI).

A further observation we have made across several cohorts is that approximately one third of individuals fail to mount a CD8 response to spike after multiple spike exposures from vaccination or infection (**Figure 4B**: 60-65% response rate for spike-specific CD8 T cells after 3-4 exposures). In preliminary analyses, we have noted the potential association of specific HLA with CD8 hyporesponsiveness **(Figure 6)**. Absence of virus-specific CD8 responses may place individuals at risk of severe outcomes, despite vaccination. Thus, we will identify BaSiS participants who fail to mount a CD8 response despite booster vaccination and combine this with HLA typing data, to identify HLA alleles enriched in non-responders. Associations with T cell hyporesponsiveness identified in BaSiS may be combined with other cohorts for further validation.

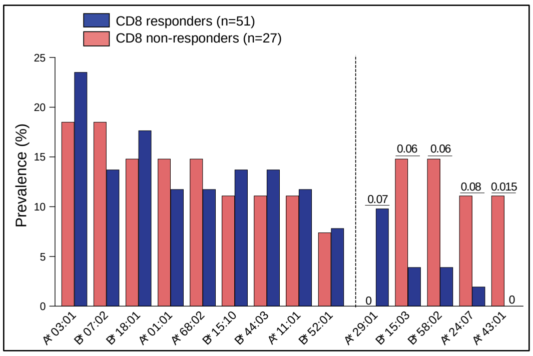

Figure 6: Prevalence of HLA alleles in spike CD8 responders and non-responders. Comparison of the distribution of the most prevalent HLA-A and B alleles in the South African population between spike CD8 responders and non-responders. Statistical comparisons were performed using a Chi test.

As part of the 6-month analysis of the BaSIS study, we are performing surveillance of T cell escape using an approach we have used previously (52-54), detecting T cell responses to variant-specific spike peptide pools. While most vaccinated or infected persons maintain highly cross-reactive T cell responses to SARS-CoV-2 variants, in preliminary work we have found that a subset of individuals have severely compromised CD8 response to variants (**Figure 7**). T cell variant cross-reactivity data generated in the first phase of the project will be combined with HLA typing data to determine whether particular Class I alleles are associated with T cell escape. Population frequency of HLA adversely affected will indicate the potential size of an escape effect. The peptide mapping approach described in **Figure 5**, combined with epitope prediction using participants' HLA, will enable us to identify individual spike mutations that result in T cell escape.

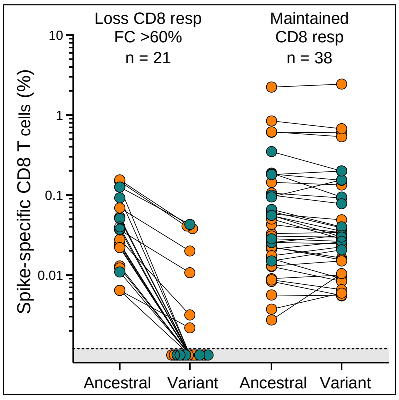

Figure 7: CD8 T cell cross-reactivity to variant spikes is lost in a proportion of individuals. Left panel: Loss of cross-reactivity of spike CD8 T cell responses tested against ancestral or Delta (teal circle) or Omicron (orange circle). Right panel: Maintenance of CD8+ T cell responses between ancestral and variant spike. FC: Fold change. An FC threshold > 60% was considered a biological difference in the response to variant spike.

6. Methodology

6.1. Trial design

BaSiS is a phase II randomised open label clinical trial in health care workers and other participants, age ≥18 years, who have previously received only one dose of the J&J Ad26.COV2.S vaccine and no other booster vaccines. The study enrolled and vaccinated 289 participants, 117 PLHIV and 36 ≥ 55 years as previously described (Table 5).

Participants were randomised 1:1:1:1 to group A, B, C and D. Group A received the Ad26.COV2.S prime plus full dose Ad26.COV2.S booster at a dose of 5x 10^{10} vp/ml (0.25 ml); Group B received the Ad26.COV2.S prime plus half dose Ad26.COV2.S booster at a dose of 2.6x 10^{10} vp/ml (0.13 ml); Group C received Ad26.COV2.S prime plus full dose BNT162b2 booster at a dose of 30 mcg (0.3 ml); and Group D received Ad26.COV2.S prime plus half dose BNT162b2 booster at a dose of 15 mcg (0.15 ml). Numbers of participants randomised to each arm are tabulated in table 4. In the primary study participants were followed up for 6 months on study after randomisation. This extension study includes additional follow-up visits at **12, 18, and 24 months** after randomisation.

6.2. Study groups for the extension study

Based on available data at 2-weeks and 3-months, participants were offered an additional booster vaccine as previously described. Participants will retain their original study group, however any additional booster vaccinations (extension booster) and breakthrough infections will be accounted for in the analysis.

Table 8: Study groups for the J&J Ad26.COV2.S/Pfizer BNT162b2 prime-boost study

Group	Number	Time after J&J Ad26.COV2.S prime	Objectives	Vaccination	Vaccine Schedule
A<br>N= 74	47 HIV Uninfected<br>27 PLHIV	≥ 4 months	Immunogenicity and safety	J&J Ad26.COV2.S<br>5x 10^{10} vp/ ml	One dose (0.25 ml)
B<br>N=69	41 HIV Uninfected<br>28 PLHIV	≥ 4 months	Immunogenicity and safety	J&J Ad26.COV2.S<br>2.6x 10^{10} vp/ ml (dose for rounded off volume)	One dose (0.13 ml – rounded off for ease of correct dosing)
C<br>N=73	42 HIV uninfected<br>31 PLHIV	≥ 4 months	Immunogenicity and safety	Pfizer BNT162b2 30 mcg IMI	One dose (0.3 ml)
D<br>N=73	43 HIV uninfected<br>30 PLHIV	≥ 4 months	Immunogenicity and safety	Pfizer BNT162b2 15 mcg IMI	One dose (0.15 ml)

As described above, all participants meeting criteria were offered a full dose Pfizer booster as part of the existing protocol, version 2.0. Currently, it is uncertain which vaccines will be available in South Africa for boosting post expiry of the Pfizer vaccines on the 31st January 2023. As part of BaSiS extension, we would like to offer an additional booster vaccine, ideally a heterologous vaccine (for example a protein-based vaccine such as Novavax), or an additional Pfizer BNT162b2 vaccine or if accessible a bivalent COVID-19 vaccine. We are engaging with various role-players to access these vaccines, however details of this are not currently available. In addition, consideration of the variant of concern at the time

of extension booster as well as emerging participant data will be included in decision making, which will be made with the study DSMB. These components will be added to the proposal as they evolve, and vaccine selection will be confirmed in a clarification memo.

Table 9: Summary of extension boosters-vaccine type and timing for proposed extension study

Product	Timing	Number participants	Contingencies	Bleed schedule
Pfizer BNT162b2 full dose	6 months post BaSiS- assigned boost vaccine (Under current protocol version 2.0)	At least 216 (87 PLWH)	Access to Pfizer BNT162b2 full dose through DoH (In progress)	No additional blood draws planned in current protocol version 2.0
Pfizer BNT162b2 full dose<br><br>OR Protein based vaccine such as Novavax<br><br>OR bivalent mRNA vaccines	6-12 months post Pfizer BNT162b2 full dose (Extension protocol 3.0)	289 participants (116 PLHIV)	Access to additional Pfizer BNT162b2 or Protein-based vaccine such as Novavax or bivalent mRNA vaccine	12 & 18 & 24 months

6.3. Trial participants

289 study participants are ≥ 18 years, 40% PLHIV and 12% ≥ 55 years (Table 4, 5).

6.4. Potential risks for participants

There are no additional risks for participants in the extension study. Risks may be associated with phlebotomy, vaccine reactogenicity, anaphylaxis, excessive response to the booster vaccine, and disclosure of HIV status.

6.4.3. Venipuncture

Bruising or swelling may occur as a result of venipuncture. Across the course of the study 70-90 ml of blood will be taken per visit (SOE Table 7), in total less than 300 ml over 18 months which is an acceptable volume according to the Office for Human Research Protections (OHRP). The recommended maximum volume of blood taken in “healthy, nonpregnant adults who weigh at least 110 pounds” is that “the amounts drawn may not exceed 550 ml in an 8 week period and collection may not occur more frequently than 2 times per week”(55).

6.4.4. Vaccine reactions to additional booster vaccines

6.4.4.1. Local reactions

May include pain, tenderness, redness or swelling at the vaccination site.

6.4.4.2. Systemic reactions

May include, but not limited to fever, malaise, tiredness, headache, fatigue, myalgia, chills, cough and loss of taste and smell, and usually last 2-3 days post booster vaccination.

With the Pfizer BNT162b2 vaccine, anaphylactic responses have also been reported worldwide as a rare event, with 175 cases of severe allergy with 21 cases (11.1/1 million) confirmed to be anaphylaxis meeting the Brighton collaboration case definition criteria (23). Myocarditis has been reported with the mRNA vaccines, at a frequency of 39–47 cases/million, more commonly in males younger than 30 years (22) with the majority of cases being self-limiting. Syncope post vaccination may also occur in rare cases.

6.4.5. Response to booster

It is possible that participants who receive the booster may have an enhanced response to the vaccine resulting in excess systemic symptoms.

6.4.6. Anaphylactic reactions

Anaphylactic reactions to the vaccines are possible but extremely rare. The clinical teams at each site are trained in advanced clinical life support (ACLS) and are able to manage and stabilise a participant who develops anaphylaxis, prior to transfer to hospital care for further observation and management.

6.4.7. Loss of privacy or confidentiality

HIV testing will be done at study screening/entry visit. An HIV test will be conducted for all participants not known to be PLHIV, and have either previously tested HIV-negative or have an unknown HIV status. PLHIV will not be re-tested for HIV. HIV testing will be offered to participants at the 12-month and 24-month visit, unless they are known to be PLHIV. In the situation where a person tests HIV positive, there is a small risk that HIV status may result in loss of confidentiality, stigma, or a lack of privacy. This will be avoided as far as possible by confidential management of all study source notes and documentation, use of a participant identifier and research staff following a strong code of principles regarding maintenance of privacy and confidentiality. PLHIV who are newly diagnosed will remain on study, be counselled about their HIV status, and be referred to HIV clinical and ART services. People living with HIV and those who are HIV-uninfected will have the same study visits conducted to minimise any potential loss of confidentiality.

6.5. Known potential benefits

This study provided one of four booster vaccine options to participants (the J&J Ad26.COV2.S homologous boost at full dose (5x 10^{10} vp/ml, 0.25 ml) and half dose (2.6x 10^{10} vp/ml, 0.13 ml) and the Pfizer BNT162b2 at full dose (30mcg, 0.3 ml) and half dose (15mcg, 0.15 ml)) ≥ 4 months after the J&J Ad26.COV2.S vaccine prime. Early interim analysis of immunogenicity data guided repeat booster vaccines and almost all participants were offered a full dose Pfizer BNT162b2 vaccine booster under evrsion 2.0 of the protocol. Although it is uncertain what vaccines will be available for appropriate boosting after January 2023, the study team is working towards access for at least an additional Pfizer vaccine at the 12-24 month visit, or other options including Novavax (protein-based) or a bivalent vaccine. We will also evaluate ongoing protection against SARS-CoV-2 infection and moderate/severe disease, although these numbers are expected to be low.

6.6. Recruitment and Withdrawal of trial participants

No additional recruitment will take place on the BaSiS Extension study.

6.7. Informed consent process

Participants will sign informed consent to continue on the extension phase of the BaSiS study for additional 12, 18 and 24 month visits.

The informed consent documents include the following:

- Main ICF
- Storage IVFICF
- HLA testing ICF
- HIV testing ICF for people who are not known to be living with HIV

Each participant will be given the opportunity to read through the ICFs. The participant will have the opportunity to ask any questions regarding the ICFs and the study with the research team and will also be able to take the ICFs home for further discussion with family or other support whilst considering participation. Comprehension will be assessed and the ICFs will be signed before any study procedures are conducted. ICFs will be offered in English, with strict adherence to GCP principles relating to the use of translators and witnesses for English-illiterate volunteers. For the purpose of this study, only English ICFs will be available.

The following principles will be emphasized during the ICF discussion:

- Participation in the study is entirely voluntary;
- Refusal to participate involves no penalty or loss of medical benefits;
- The participant has the right to withdraw participation at any time;
- The participant may ask questions at any time to increase understanding of the purpose of the study and the procedures involved;
- The study is investigating the durability of responses to the four dosing approaches to COVID-19 vaccine boosting of the J&J Ad26.COV2.S vaccine given through the SISONKE phase IIIB implementation study or the South African Covid-19 vaccination programme, namely the J&J Ad26.COV2.S vaccine (5x 10^{10} vp/ml) or half dose (2.6x 10^{10} vp/ml) or the Pfizer BNT162b2 vaccine at 30mcg or 15mcg doses; with an additional Pfizer full dose booster recommended by the DSMB as discussed above;
- Since a booster vaccine is being given to participants as part of protocol version 2.0 as discussed, as well as an additional extension booster on the extension protocol, there is an expected direct benefit to participating in the study;
- Participants will be asked to provide detailed medical and surgical history to investigator verbally and if possible, patient-held medical records (outpatient cards) will be reviewed;
- Documentation proving the identity of the participant will be required and confidentiality will be maintained;
- A Biometric Co-Enrolment Prevention System or similar process will be used by all sites however some co-enrolment in other interventional studies will be permitted on a case-by-case basis and the ethics committee will be notified if this occurs.

6.8. Inclusion and exclusion criteria

6.8.1. Inclusion criteria

All inclusion criteria for the study must be met for participation:

1. All participants vaccinated in the primary BaSiS study, completed 6 months of follow up, willing to continue on study and attend 12, 18 and 24 month visits will be eligible for the BaSiS extension study.

2. Willing and able to sign informed consent.
3. Able in the investigator's opinion to comply with study procedures, including willingness to be tested for HIV and to attend Covid-19 illness visits, if applicable.
4. Plan to stay within reasonable distance of the study site to attend study visits.

6.8.2. Exclusion criteria

Not willing to continue participation in the BaSiS extension study.

A non-study Covid-19 vaccine received at any time is not exclusionary. These participants will have the non-study vaccine noted on source and data may be analysed separately.

Previous COVID-19 infection is not exclusionary.

6.8.3. Female participants of childbearing potential

For the extension study, a negative pregnancy test may be required when additional booster doses are offered to participants, depending on which booster option is offered and depending on available data for that vaccine in pregnancy. A pregnancy test will be conducted in WOCP at the vaccination visit and the pregnancy followed up for safety and outcome. National guidelines from Department of Health recommend vaccination for all pregnant women with either of the currently available vaccines.

6.9. Trial procedures

All participants will attend study scheduled visits until 24 months post-randomisation and initial booster vaccination.
Study visit procedures for HIV-uninfected and PLHIV participants are tabulated in Table 7. Study visits occur at screening and enrolment, 2 weeks, 3 months, 6 months, 12 months, 18 months and 24 months. Blood samples at each study visit will be taken, up to approximately 70 ml per visit, described in Table 7, SOE.

6.9.1. Observations

At each visit, vital signs including temperature, heart rate, respiratory rate, oxygen saturation on room air and blood pressure will be measured. A medical history will be taken including questions regarding recent COVID-19 close contact, COVID-19 symptoms, positive SARS-CoV-2 antigen or nasopharyngeal polymerase chain reaction test and a targeted physical examination conducted if necessary.

6.9.2. Spike binding antibody assays

Spike binding antibody assays will be performed at enrolment, 2 weeks, 3 months, 6, 12, 18 and 24 months. Assays will be performed as follows: 2mcg/ml of ancestral D614G spike protein will be used to coat 96-well, high-binding plates and incubated overnight at 4°C. The plates will be incubated in a blocking buffer consisting of 5% skimmed milk powder, 0.05% Tween 20, 1x PBS. Plasma samples will be diluted to 1:100 starting dilution in a blocking buffer and added to the plates. Secondary antibody will be diluted to 1:3000 in blocking buffer and added to the plates followed by TMB substrate (Thermofisher Scientific). Upon stopping the reaction with 1M H_2SO_4, absorbance will be measured at a 450nm wavelength. In all instances, mAbs CR3022 and BD23 will be used as positive controls and palivizumab was used as a negative control. All values will be normalized with the CR3022 mAb(58).

6.9.3. Neutralisation Assays

6.9.3.1. Pseudovirus neutralisation assays

These will be performed on spike positive samples at enrolment, 2 weeks, 3 months, and 6 months. SARS-CoV-2 pseudotyped lentiviruses will be prepared by co-transfecting the HEK 293T cell line with either the SARS-CoV-2 ancestral variant spike (D614G), the Beta spike (L18F, D80A, D215G, K417N, E484K, N501Y, D614G, A701V, 242-244 del) or the Delta spike (T19R, R158G L452R, T478K, D614G, P681R, D950N, 156-157 del) plasmids in conjunction with a firefly luciferase encoding lentivirus backbone plasmid. As other VOC emerge these may be included in this panel. For the neutralisation assay, heat-inactivated plasma samples from vaccine recipients will be incubated with the SARS- CoV-2 pseudotyped virus for 1 hour at 37°C, 5% CO_2. Subsequently, $1x10^4$ HEK 293T cells engineered to over-express ACE-2 will be added and incubated at 37°C, 5% CO_2 for 72 hours upon which the luminescence of the luciferase gene was measured. CB6 will be used as a positive control.

6.9.3.2. Live virus neutralisation assays

These will be performed at enrolment, 2 weeks, 3 months and 6 months. Vero E6 cells will be plated in a 96-well plate at 30,000 cells per well 1 day before infection. Plasma will be separated from EDTA-anticoagulated blood by centrifugation at 500 rcf for 10 minutes and stored at −80°C. Aliquots of plasma samples will be heat-inactivated at 56°C for 30 minutes and clarified by centrifugation at 10,000 rcf for 5 minutes, after which the clear middle layer will be used for experiments. Inactivated plasma will be stored in single-use aliquots to prevent freeze–thaw cycles. For experiments, plasma will be serially diluted twofold from 1:100 to 1:3,200. As a positive control, the GenScript A02051 anti-spike monoclonal antibody will be added. Virus stocks will be used at approximately 100 focus-forming units per microwell and added to diluted plasma; antibody–virus mixtures will be incubated for 1 hour at 37°C, 5% CO_2. Cells will be infected with 100µl of the virus–antibody mixtures for 1 hour, to allow adsorption of virus. Subsequently, infection will be overlaid with 1.5% carboxymethylcellulose, Infection will be fixed with 4% PFA after 24 hours and imaged using an Elispot reader or automated microscope. SARS-CoV-2 variants will include sequenced outgrowths of ancestral SARS-CoV-2 with D614G only, beta, delta, and new variants to emerge.

6.9.4. T cell assays

6.9.4.1. Cell stimulation and flow cytometry staining

Cryopreserved PBMC will be thawed, washed and rested in RPMI 1640 containing 10% heat-inactivated FCS for 4 hours prior to stimulation. PBMC will be seeded in a 96-well V-bottom plate at ~2×10^6 PBMC per well and stimulated with SARS-CoV-2 spike peptide pool based on the full spike protein (Miltenyi Biotec), at 1µg/ml. A subset of samples will be tested for cross-reactivity of T cells to beta, delta, and new variants to emerge, using peptide pools based on these sequences. All stimulations will be performed in the presence of Brefeldin A (10µg/ml, Sigma-Aldrich, St Louis, MO, USA) and co-stimulatory antibodies against CD28 (clone 28.2) and CD49d (clone L25) (1µg/ml each; BD Biosciences, San Jose, CA, USA). As a negative control, PBMC will be incubated with co-stimulatory antibodies, Brefeldin A and an equimolar amount of DMSO.

After 16 hours of stimulation, cells will be washed, stained with LIVE/DEAD™ Fixable VIVID Stain (Invitrogen, Carlsbad, CA, USA) and subsequently surface stained with the following antibodies: CD14 Pac Blue (TuK4, Invitrogen Thermofisher Scientific), CD19 Pac Blue (SJ25-C1, Invitrogen Thermofisher Scientific), CD4 PERCP-Cy5.5 (L200, BD Biosciences, San Jose, CA, USA), CD8 BV510

(RPA-8, Biolegend, San Diego, CA, USA), CD27 PE-Cy5 (1A4, Beckman Coulter), CD45RA BV570 (HI100, Biolegend, San Diego, CA, USA). Cells will be fixed and permeabilized using Cytofix/Cytoperm buffer (BD Biosciences) and stained with CD3 BV650 (OKT3), IFN-g Alexa700 (B27), TNF BV786 (Mab11) and IL-2 APC (MQ1-17H12) from Biolegend. Finally, cells will be washed and fixed in CellFIX (BD Biosciences). Samples will be acquired on a BD LSR-II flow cytometer and analyzed using FlowJo (v10, FlowJo LLC, Ashland, OR, USA). Cells will be gated on singlets, CD14- CD19-, live lymphocytes and memory cells (excluding naive CD27+ CD45RA+ population). Results will be expressed as the frequency of CD4+ or CD8+ T cells expressing IFN-g, TNF-a or IL-2. Parallel stimulation cultures may be set up in the absence of BFA and supernatant harvested for IFN-g ELISA.

6.10. Study visits

6.10.1. Screening and enrolment visit (Day 0), 2-week, 3-month and 6-month visits (Participants off study)

These visits were completed in version 2.0 of the protocol, and procedures are summarised in the SOE, table 7 (non-bold).

6.10.2. Month 6 visit (±14 days) (Participants still on study)

- Vitals
- Medical and COVID-19 history
- Concomitant medication review
- Targeted physical examination if required
- Immunology bloods
- Immunogenicity bloods
- People with an inadequate response to the booster will be offered a full dose Pfizer vaccine based on study findings previously described, if not already received
- ICF for the BaSiS Extension study
- Eligibility assessment for BaSiS extension

6.10.3. Repeat booster visit (6 months +4 months) (Participants completed study)

- As described, based on interim results and DSMB adjudication, participants who meet study criteria for a repeat booster (all J&J arms, half dose Pfizer arm, and those who received a full dose Pfizer vaccine >6 months previously), will be contacted offered a full dose Pfizer booster under version 2.0 of the protocol. This timing may vary by site due to staggered enrolment but will ideally occur at 6 months post enrolment.

6.10.4. Month 12 visit (Participants completed study)

- Voluntary written informed consent for participation in the extension study, as well as for HLA testing, HIV testing for people who are previously HIV-negative and sample storage will be obtained prior to commencement of study procedures.
- Eligibility check

- Once ICF is obtained, vital signs will be measured, including blood pressure, pulse rate, oxygen saturation in room air, respiratory rate, oral temperature with a plastic sleeve over the thermometer or tympanic temperature with an infrared tympanic thermometer. Weight and height will be measured, and body mass index calculated.
- Medical history will be reviewed and updated as required.
- Concomitant medication taken since month 6 visit will be reviewed and updated, including receipt of any additional off-study COVID-19 vaccines.
- COVID-19 history will be taken including history of previously confirmed SARS-CoV-2 infection by nasopharyngeal PCR or antigen testing, or a positive SARS-CoV-2 antibody test; close contact diagnosed with SARS-CoV-2 infection; symptoms since March 2020 that could be attributed to SARS-CoV-2 infection (including but not limited to fever or chills; cough; shortness of breath or breathing difficulties; fatigue; muscle or body aches; headache; loss of taste or smell newly developed; sore throat; congestion or runny nose; nausea or vomiting and diarrhoea).
- Targeted physical examination, if necessary.
- Urine pregnancy test in WOCP, if vaccinating at this visit (see section 6.8.3).
- HIV Elisa test in people who are not known to be living with HIV.
- Participants who are not known to be living with HIV and who test positive for HIV at this visit will receive counselling from a trained staff member. They will be referred to a practitioner or clinic of their choice for further management and for initiation of ART.
- Immunology blood tests and HIV viral load in PLHIV (as described in the SOE Table 7).
- Immunogenicity bloods as described in SOE Table 7.
- Nasopharyngeal swab
- Vaccination and post-vaccination observations, if vaccinating at this visit (extension booster) (see section 6.10.7)

6.10.5. Month 18 visit

- Vitals as previous
- Medical and COVID-19 history as previously described.
- Concomitant medication review including additional COVID-19 vaccinations
- Targeted physical examination if required
- Urine pregnancy test in WOCP, if vaccinating at this visit (see section 6.8.3).
- Immunology bloods and HIV viral load in PLHIV
- Immunogenicity bloods
- Nasopharyngeal swab
- Vaccination and post-vaccination observations, if vaccinating at this visit (extension booster) (see section 6.10.7)

6.10.6. Month 24 visit

- Vitals as previous
- Medical and COVID-19 history as previously described.
- Concomitant medication review including receipt additional COVID-19 vaccinations.
- Targeted physical examination, if necessary.

- Urine pregnancy test in WOCP, if vaccinating at this visit (see section 6.8.3).
- HIV Elisa test in people who are not known to be living with HIV.
- Participants who are not known to be living with HIV and who test positive for HIV at this visit will receive counselling from a trained staff member. They will be referred to a practitioner or clinic of their choice for further management and for initiation of ART.
- Immunology blood tests and HIV viral load in PLHIV (as described in the SOE Table 7).
- Immunogenicity bloods as described in SOE Table 7.
- Nasopharyngeal swab
- Vaccination and post-vaccination observations, if vaccinating at this visit (extension booster) (see section 6.10.7)

6.10.7. Booster vaccination visit 12-24 months

- At either one of the extension visits between 12-month and 24-months, approximately 6 months after the repeat Pfizer booster, participants will be offered an additional booster vaccine if this is available. Potential options are tabulated in Table 9 and will be confirmed with a clarification memo as soon as confirmation is available. The selected options all demonstrate good immunogenicity as a homologous or heterologous booster.
- Contraceptive history and urine pregnancy testing for all female participants of child-bearing potential on the day of vaccination.
- Vaccination.
- Observation for at least 15 minutes post vaccination, including vital signs and assessment of local and systemic reactions.
- Participants will be asked to contact the study site if they have any ongoing solicited reactogenicity at day 7-post vaccination, any unsolicited AEs up to 28 days post vaccination and any SAEs thoughout the study.

6.10.8. Interim/Illness visits - only if indicated

Interim visits will be conducted for the following reasons:

- Reactogenicity event greater or equal to grade 3 or any reactogenicity event ongoing at day 7.
- Solicited AEs beyond 7 days post vaccination, or unsolicited AEs throughout the study post vaccination if they are grade 3 or higher.
- Intercurrent SARS-CoV-2 symptoms or confirmed infection at any of the extension study visits, or after testing by a non-study provider.

The participant will contact site staff who will then arrange the interim visit.

For participants requiring an interim visit the following procedures will be performed:

- Vitals
- Medical and COVID-19 history (previously confirmed SARS-CoV-2 infection by NP PCR or antigen testing, or a positive SARS-CoV-2 antibody test; close contact diagnosed with SARS-CoV-2 infection; symptoms since March 2020 that could be attributed to SARS-CoV-2 infection (including but not limited to fever or chills; cough; shortness of breath breathing difficulties; fatigue; muscle or body aches; headache; loss of taste or smell newly developed; sore throat; congestion or runny nose; nausea or vomiting and diarrhoea).

- Concomitant medication review.
- Illness visits will be conducted as soon as possible after the participant experiences symptoms or reports to have tested positive for SARS-CoV-2 infection. Management of SARS-CoV-19 disease will be the responsibility of the participant's attending doctor or clinic and the site will not take over management of these episodes. The participant will be seen once in an infection-controlled area within the clinic
- AE review
- Targeted physical examination
- SARS-CoV-2 nasopharyngeal PCR test (illness visits)

7. Investigational Product

The investigational product in the study includes the following:

- Johnson and Johnson adenovirus vector vaccine Ad26.COV2.S at full (5.0 x 10^{10}) and half (2.6 x 10^{10}) dose.
- Pfizer BNT162b2 mRNA vaccine by Pfizer BioNTech at full (30mcg) and half (15mcg) dose.

Participants will be randomised to one of four groups. Group A and B will receive the Johnson and Johnson adenovirus vector vaccine. Group C and D will receive the Pfizer mRNA vaccine. Each group will be assigned the investigational product at full, and half dose as detailed in the table 10 below:

Table 10: Reconstitution of vaccine in primary BaSiS study

	Group A	Group B	Group C	Group D
**Type of Vaccine**	Janssen Ad26.COV2.S		Pfizer BNT162b2	
**Formulation**	Adenovirus vector vaccine		mRNA vaccine	
**Dosage**	5.0x 10^{10} vp/ ml	2.6 x 10^{10} vp/ ml	30mcg	15mcg
**Diluent**	None required		0.9% Sodium chloride Injection USP. **No bacteriostatic 0.9% Sodium chloride should be used**	
**Final volume to be administered**	0.25 ml	0.13 ml	0.30 ml	0.15 ml
**Maximum doses per vial**	2	4	6	12
**Route of administration**	Intramuscular, deltoid muscle of non-dominant arm			

7.1. J&J Ad26.COV2.S adenovector vaccine

The J&J vaccine is composed of recombinant, replication-incompetent human adenovirus type 26 vector that, after entering human cells, expresses the SARS-CoV-2 spike S antigen without virus propagation. An immune response elicited to the S antigen protects against COVID-19. The vaccine will be supplied by SAMRC/Janssen as a frozen, multidose, preservative free suspension (0.50 ml).

7.2. Pfizer BioNTech BNT162b2 modRNA vaccine

The Pfizer vaccine is formulated in lipid particles that enable delivery of the RNA into the host cells. The host cells allow expression of the SARS-CoV-2 S antigen. The vaccine elicits an immune response to the S antigen which protects against COVID-19. The vaccine will be supplied by the Department of Health as a frozen, multidose, preservative free suspension (0.45 ml). Each vial will require dilution with 0.9% sterile Sodium chloride (1.80 ml).

Refer to the investigational product (IP) manual for further clarification on study product.

7.3. The Covovax^{TM} vaccine

The Covovax^{TM} vaccine is a protein subunit vaccine consisting of recombinant SARS-COV-2 spike protein fragments assembled into stabilised nanoparticles. The nanoparticles assist the body's immune system in creating antibodies which respond to SARS-COV-2 infection. The vaccine contains a saponin-based adjuvant; Matrix-M that activates cells of the immune system, generating a more powerful response. The vaccine will be supplied by Serum Institute of India, if we are able to access it for this study. The vaccine is either supplied in a single dose vial (0.5mL) or a multidose vial (10 doses in 5mL). The vaccine must be stored in a refrigerator (2°C to 8°C) and must be protected from light. The Covovax^{TM} vaccine will be given to participants as the extension study booster as described dependant on availability and VOC.

7.4. IP preparation, handling, storage and accountability

7.4.1. J&J Ad26.COV2.S adenovector vaccine

The J&J vaccine is supplied as multi dose vials in cartons of 20. The cartons will arrive as frozen. Vials should be stored in a freezer between -15°C and -25°C. Vials must be kept frozen and protected from light. Vials must be kept in original cartons in the freezer in an upright position.

7.4.2. Pfizer BioNTech BNT162b2 modRNA vaccine

The Pfizer BioNTech vaccine (COMIRNATY®) is supplied as multi dose vials in cartons of 25 or 195. The cartons will arrive in thermal containers with dry ice. Vials should be stored in an ultra-low freezer between -90°C to -60°C. Vials must be kept frozen and protected from light during storage.

The diluent (0.9% Sodium chloride injection USP) is provided separately and should be stored at room temperature (20-25°C). The provided diluent will be supplied as either 10 ml or 2 ml single use vials.

For both vaccines, the site pharmacist or designated study personnel will confirm if the study product has arrived at the appropriate temperature range as stipulated in the IP manual. The study product should be stored on site in a secure location and at the correct temperature according to manufacturers' recommendations, with limited access to unauthorized persons and at controlled temperatures as indicated on the clinical labels. Any temperature excursions should be reported as described in the IP manual.

Study vaccines will be prepared by appropriately qualified and delegated study personnel, and in accordance with the IP manual. Site pharmacists will be responsible for all study product accountability. All product received and dispensed must be accounted for using site-specific SOPs and accountability logs.

7.5. IP destruction

The process of destruction of any unused study product and documentation of destruction is detailed in the IP manual and should be followed accordingly.

8. Assessment of safety

Safety will be assessed according to frequency, severity, and type of adverse event and severe adverse event. Local and systemic solicited and unsolicited reactogenicity adverse events will be collected following booster vaccine on day 0 until 28 days after vaccination.

The following solicited reactogenicity AEs will be collected if still experienced by the participant 7days post extension booster vaccination:

- Pain
- Tenderness
- Erythema/Redness
- Induration/Swelling
- Fever > 38.0 degrees Celsius
- Nausea/Vomiting
- Diarrhoea
- Headache
- Fatigue
- Myalgia
- Chills
- Cough
- Loss of taste and/or smell

SAEs and AESI will be collected from enrolment until study end, as well as any AEs resulting in withdrawal of the participant from the study.

8.1. Definitions

8.1.1. Adverse Event (AE)

Any untoward medical occurrence that occurs during the time of administration of a study product, including events related and not related to the study product.

8.1.2. Adverse reaction (AR)

This is a reaction to the study product assessed to be related (definitely, probably or possibly) to the study product as evaluated by the investigator.

8.1.3. Serious Adverse event (SAE)

This is an adverse event that results in one of the following outcomes:

- Death
- Life-threatening if no intervention
- Hospitalisation or prolonged hospitalisation
- Results in permanent or significant disability
- Congenital anomaly

8.1.4. Important medically attended event

This is an adverse event that does not meet criteria for an SAE, SUSAR or AESI but is a medical visit for a reason other than routine study visit or vaccination and requires a hospital, emergency room or unscheduled visit to medical personnel (including the site) for any reason.

8.1.5. Suspected Unexpected Severe Adverse Reaction (SUSAR)

A suspected unexpected serious adverse response to the study product, not included in the study product information brochure but meeting the criteria as defined in "Serious adverse event". Since no SAEs are expected as a result of booster vaccination in this study, all SAEs related to vaccine will be reported as SUSARs.

8.1.6. Adverse Event of Special Interest (AESI)

AESIs will include the following AEs or SAEs:

- Moderate or severe SARS-CoV-2 infection resulting in hospitalisation, death or where residual complications occur such as oxygen dependency.
- Myocarditis, stroke or any other neurological complications of SARS-CoV-2 infection.

8.2. Grading of events

The Guidance for Industry: Toxicity Grading Scale for Healthy Adult and Adolescent Volunteers Enrolled in Preventive Vaccine Clinical Trials (59) will be the primary grading table in keeping with the previous Pfizer COVID vaccine grading standards. For reactogenicity that remains beyond 7-days post extension booster vaccination, participants will be seen at site and events will be graded as below.

8.2.1. Reactogenicity

Evaluated after 15 minutes post-vaccination and then if solicited or related unsolicited AEs are still present 7-days post vaccination.

8.2.1.1. Local reactions

Expected reactions include pain, tenderness, erythema/redness and induration/swelling. These local reactions will be graded according to FDA guidance below (59):

**Local Reaction to Injectable Product**	**Mild (Grade 1)**	**Moderate(Grade 2)**	**Severe (Grade 3)**	**Potentially Life Threatening (Grade 4)**
Pain	Does not interfere with activity	Repeated use of non-narcotic pain reliever > 24 hours or interferes with activity	Any use of narcotic pain reliever or prevents daily activity	Emergency room (ER) visit or hospitalization
Tenderness	Mild discomfort to touch	Discomfort with movement	Significant discomfort at rest	ER visit or hospitalization
Erythema/Redness *	2.5 – 5 cm	5.1 – 10 cm	> 10 cm	Necrosis or exfoliative dermatitis
Induration/Swelling **	2.5 – 5 cm and does not interfere with activity	5.1 – 10 cm or interferes with activity	> 10 cm or prevents daily activity	Necrosis

f8.2.1.2. Systemic reactions

Systemic reactions including fevers above 38.0°C, nausea, vomiting, diarrhoea, headache, fatigue myalgia, chills, cough, loss of taste or smell will be recorded on the diary card.

Anaphylaxis will be graded according to criteria included in Appendix 1.

Systemic reactions will be graded as follows (FDA) if ongoing day 7 post vaccination.

**Systemic (General)**	**Mild (Grade 1)**	**Moderate(Grade 2)**	**Severe (Grade 3)**	**Potentially Life Threatening (Grade 4)**
Nausea/vomiting	No interference with activity or 1 – 2 episodes/24 hours	Some interference with activity or > 2 episodes/24 hours	Prevents daily activity, requires outpatient IV hydration	ER visit or hospitalization for hypotensive shock
Diarrhea	2 – 3 loose stools or < 400 gms/24 hours	4 – 5 stools or 400 – 800 gms/24 hours	6 or more watery stools or > 800gms/24 hours or requires outpatient IV hydration	ER visit or hospitalization
Headache	No interference with activity	Repeated use of non-narcotic pain reliever > 24 hours or some interference with activity	Significant; any use of narcotic pain reliever or prevents daily activity	ER visit or hospitalization
Fatigue	No interference with activity	Some interference with activity	Significant; prevents daily activity	ER visit or hospitalization
Myalgia	No interference with activity	Some interference with activity	Significant; prevents daily activity	ER visit or hospitalization

**Systemic Illnes**	**Mild (Grade 1)**	**(Moderate(Grade 2)**	**Severe (Grade 3)**	**Potentially Life Threatening (Grade 4)**
Illness or clinical adverse event (as defined according to applicable regulations)	No interference with activity	Some interference with activity not requiring medical intervention	Prevents daily activity and requires medical intervention	ER visit or hospitalization

8.2.2. Vital signs

Vital signs will be graded according to the FDA grading tables below.

Vital Signs *	Mild (Grade 1)	Moderate(Grade 2)	Severe (Grade 3)	Potentially Life Threatening (Grade 4)
Fever (°C) **<br>(°F) **	38.0 – 38.4<br>100.4 – 101.1	38.5 – 38.9<br>101.2 – 102.0	39.0 – 40<br>102.1 – 104	> 40<br>> 104
Tachycardia - beats per minute	101 – 115	116 – 130	> 130	ER visit or hospitalization for arrhythmia
Bradycardia - beats per minute***	50 – 54	45 – 49	< 45	ER visit or hospitalization for arrhythmia
Hypertension (systolic) - mm Hg	141 – 150	151 – 155	> 155	ER visit or hospitalization for malignant hypertension
Hypertension (diastolic) - mm Hg	91 – 95	96 – 100	> 100	ER visit or hospitalization for malignant hypertension
Hypotension (systolic) – mm Hg	85 – 89	80 – 84	< 80	ER visit or hospitalization for hypotensive shock
Respiratory Rate – breaths per minute	17 – 20	21 – 25	> 25	Intubation

* Subject should be at rest for all vital sign measurements.
** Oral temperature; no recent hot or cold beverages or smoking.
*** When resting heart rate is between 60 – 100 beats per minute. Use clinical judgement when characterizing bradycardia among some healthy subject populations, for example, conditioned athletes.

8.3. Causality

The site investigator will assess the relationship between the study vaccine and the AE and provide an assessment of relatedness. It must be determined whether a reasonable possibility exists that the vaccine caused or contributed to a SAE.

This is based on clinical judgement and factors include:

- Whether a temporal relationship existed between the event and receipt of the vaccine.
- Whether there is a plausible biological mechanism for the vaccine to have caused the AE.
- If there is any other possible cause for the AE.
- Whether similar previous reports of AEs with the vaccine have been recorded and reported.

The AE may be assessed to be *related* (definite, probable, possible) if it is possible that the vaccine caused the AE and *not related* (not related or not likely) if there is no reasonable possibility that the vaccine caused the AE.

If an investigator assesses the AE as “not related”, an alternative cause, diagnosis, or explanation must be provided. Causality must be re-assessed when new information is available and may require review and update of the AE if applicable.

8.4. COVID BTI's will be graded as described below.

Outcome	Description	Definition
**Mild disease**	Asymptomatic	Lab confirmed COVID-19 with no symptoms
	Symptomatic	Managed at home, did not require oxygen
**Moderate disease**	Hospitalised; no oxygen therapy	Hospitalisation does not include the following:<br>- under 24 hours,<br>- unrelated to COVID-19 infection;<br>- unable to safely isolate at home
	Hospitalised; oxygen by mask/nasal prongs	In keeping with hospitalisation definition above: i.e. the following would still be considered as mild: hospitalisations under 24 hours; incidental COVID-19 infections that do not prolong hospital stay but oxygen required for primary diagnosis; etc
	Hospital at home care	Oxygen supplementation at home
**Severe disease**	Oxygen by NIV/high flow	
	Intubation & mechanical ventilation<br>$pO_2/FiO_2 \geq 150$ or $SpO_2/FiO_2 \geq 200$	
	Mechanical ventilation<br>$pO_2/FiO_2 < 150$ or $SpO_2/FiO_2 < 200$ **or** vasopressors	
	Mechanical ventilation $pO_2/FiO_2 < 150$ **and** (vasopressors, dialysis or ECMO)	
**Dead**	Dead	

8.5. Reporting of AEs

Interim visits will be scheduled for participants who have any solicited or related unsolicited AEs at day 7 post vaccination with the extension booster. In addition, any grade 3 or higher unsolicited AE within 28 days of vaccination and any SAE or grade 3 or higher AE will result in an interim visit being scheduled. Participants will be requested to notify site in such circumstances.

SAEs will be reported to the study team within 24 hours of site notification, as well as SUSARS, ADRs and AESI.

SAEs assessed to be related to study product or the trial or resulting in hospitalisation or death of a participant will be reported to the relevant ethics committee(s) within stipulated timelines, and regulatory authority (SAHPRA) according to the following guidance(60):

- Deaths or life threatening, related and unexpected reactions to be reported within 7 days (preliminary)
- New information impacting on risk-benefit profile of product or conduct of trial must be reported within 3 days.
- Other important safety info within 15 days.

9. Data Management and Analysis

9.1. Randomisation Procedure

- All participants, who enrolled in the trial were individually randomised to one of four groups:
  Group A: full dose J&J Ad26.COV2.S (5x 10^{10} vp/ml, 0.25 ml)
  Group B: half dose J&J Ad26.COV2.S (2.6x 10^{10} vp/ ml, 0.13 ml)
  Group C: full dose BNT162b2 vaccine (30mcg)
  Group D: half dose (15mcg) Pfizer BNT162b2 vaccine
- 289 participants were included across 4 arms (A = 74, B = 69, C = 73 and D = 73), . Overall 173 HIV-uninfected and 116 PLHIV were enrolled onto the study.
- 36 (12.5%) participants are ≥ 55 years of age.
- Randomisation was blinded; open label vaccination was followed.
- Stata 15 (Statacorp LLC, College Station, Texas) was used to perform stratified randomisation. Per site, randomisation was performed separately within each stratum of HIV status to achieve treatment assignment balance.
- Participants in each stratum were allocated on a 1:1:1:1 ratio in masked block sizes, ensuring balance between the groups. The study statistician or epidemiologist created the randomisation allocation lists for each group prior to study start to ensure that the correct number of participants were randomised to each group at each site.
- The randomisation allocation list was uploaded to REDCap for electronic implementation. REDCap applied the random allocation table and assigned the participant to a study arm. This study arm is shown on the REDCap Demographic Form. Following allocation, the study site and HIV status fields were locked for future editing.
- Apart from the study Epidemiologist, who created the randomisation allocation list, no other study staff has access to this allocation list, which substantially minimised the chances of being able to determine the allocation prior to enrolment.

9.2. Sample size

For the original study, we calculated the sample size necessary to detect an increase in antibody mediated neutralisation when an increase is present following booster vaccination with Pfizer BNT162b2 vaccine 15mcg IMI (half-dose) with 90% probability. Effect size for the calculation is fold-increase in neutralisation post-boost. Fold-change is assumed to be at least as large as observed with a homologous boost with a fractional dose of Ad26.COV2.S, which is 6 to 7.7-fold (15). Non-inferiority of a heterologous boost with a fractional Pfizer BNT162b2 dose is likely, due to the higher neutralisation response to this vaccine relative to Ad26.COV2.S (16).

Since this was a Phase II study with a fixed sample size of n=75 per prime/boost combination, the following effect sizes were illustrative for the study. For the primary outcome, the change in neutralising antibodies between baseline and 2-weeks post boost, the study, with n=75 participants per arm, would have 90% power to demonstrate a 4-fold increase in response at a 2.5% significance level for a paired t-test using a superiority margin of 0. For 2-fold increase superiority margin the study, with n=75 participants per arm, we had 80% power for a 6-fold increase in neutralising antibodies at 2 weeks post boost for a paired t-test at a 2.5% significance level. The baseline sample size information was based on South African laboratory data of 84 participants of which 51 were vaccinated and infected with Covid and 33 vaccinated uninfected with Covid. For the PRNT50 assay the log10(GMT) of 1.89 with standard

deviation of 1.246 was obtained. The correlation between baseline and 2-weeks post boost values was taken as 0.2 resulting in standard deviation estimate of the difference on the log10 scale of 1.474.

9.3. Database development

A web-based database was developed using REDCap (Research Electronic Data Capture), a secure, web-based application designed to support data capture for research studies (61). REDCap is a 'closed' system, which cannot be accessed without an individual username/password. There is SSL (Secure Sockets Layer) encryption, firewalls and separate application, and database servers, with connections between the two strictly regulated. Data is backed up on a separate physical machine, as well as running frequent offsite backups. Data collected at facilities are not stored on local devices (laptops/tablets). All data are immediately uploaded to the REDCap server via a secure internet connection provided by the respective study sites – public or facility-based wi-fi or other connections will not be used.

For the extension phase of the study, new data collection instruments for the new study events will be added to the existing REDCap database by the study's national data manager and epidemiologist.

REDCap has a fully traceable audit trail. High-level users may have access to view a log of all occurrences of data exports, design changes, record creation, updating & deletion, user creation, record locking, and page views. As such, any activity on the database can be traced back to an individual user.

All REDCap database users have been allocated specific roles that allow for defined access levels. This will continue for new database users. For example: a data capturer is able to create and edit participant data but is not able to delete any records, export any data or adjust user rights. Data managers have been granted higher level access to allow him/her to create users, edit fields, delete records and perform data exports under the supervision of the epidemiologist. Study PIs have full access to all modules of the database. These roles have been defined by the epidemiologist and study data manager in consultation with the study PIs.

All REDCap users are trained in GCP, Human Subjects Protection, and REDCap security. No staff member is allowed to share access credentials as they will then become liable for any activity logged against their user profile.

9.4. Data Entry and Management

9.4.1. Study-related Forms

Electronic forms were developed directly on the REDCap database including the following:

- Demographic information form
- Case report forms (CRF) for each study visit (screening and enrolment, week 2, month 3, month 6, month 12, month 18, month 24, interim and illness visit
- Medical history log
- AE log, MAAE log, SAE/AESI log, PD log
- Concomitant medication logs
- Diary card capture
- Laboratory Results
- Study disposition form
- Pregnancy on study form

Source notes were created for each CRF as above.

9.4.2. Data Entry and Processing

- A single unique participant identifying number was allocated to each participant once the ICF was signed.
- This unique identifier was used for all source documentation, all laboratory tests and all data entry.
- The participant identifying number was entered on the Screening and Enrolment Log.
- Linkage of the unique participant identifier to the participant file number and facility was recorded on the Screening and Enrolment Log. During data capture, research assistants, the data manager, and Wits RHI sub-investigators have access to the data in order to capture data and to maintain data quality.
- The database is updated with information from source documentation which contains no personally identifying information. All these documents are located in the participant's file at the specific research site, which is stored under restricted access to study personnel. All information entered in the database, is entered under the unique participant identifying number.
- All data is entered directly onto the REDCap database from the source documents.
- The database:
  - Accepts only variables within reasonable pre-defined ranges (e.g., CD4 0-2,000/µL).
  - Detects and prevents entry of inconsistencies (e.g., date of birth after date of HIV clinic enrolment).
  - Detects missing data fields and prevents omission of crucial data (e.g., missing date of ART initiation).
- All data is stored on the online REDCap database and is backed up on the Wits RHI server periodically; both of which can be accessed only using password-protected logins.
- Only the data manager and the study investigators have access to accumulating data across all sites.

9.4.3. Data Security

- Data security systems comply with the requirements of the POPIA Act.
- All sites ensure that antivirus software safeguards all devices and systems from viruses and other malware.
- All handheld devices have all information wiped from memory and returned to factory settings at the end of the data collection process.
- Inoperable devices will have all information wiped from memory and returned to factory settings (if possible).
- Using REDCap databases further ensures that no participant information is stored on local devices.
- Data exported from REDCap for analysis purposes is de-identified. This data is stored on Wits RHI's OneDrive/SharePoint server that includes encryption linked to Authorised Directory user authentication, i.e., a user is not able to access another user's OneDrive unless login details are shared. Sharing of login details is not permitted under Wits RHI's IT policy.

9.4.4. Data Quality Control

On-site quality control (QC) is performed by delegated staff according to the site SOP to ensure that the study is proceeding according to study protocols and Good Clinical Practice (GCP) requirements, the

personnel are fulfilling their job descriptions and responsibilities, the data collected is accurate and complete, and confidentiality of participants is observed.

The respective site Data Manager/s are responsible for ensuring that data quality is maintained and review collected data on a weekly basis to ensure that all fields are collected appropriately in the correct format.

Data quality attributes that are checked:

- Within range values
- Missing data
- Data formats

When discrepancies are noted, queries are raised with the clinical staff (clinicians, study coordinators and nurses) and corrections are completed on source documents and on the REDCap database. All errors are corrected in real time in the source as they are discovered, and on the data base within 72 hours of source correction. A log is kept of all changes and corrections.

9.4.5. Data Transfer

- Tablet/Laptop devices are accessed using password-protected logins and will communicate with REDCap using SSL (Secure Sockets Layer), a security technology for establishing an encrypted link between a web server and a browser. All data is immediately uploaded onto the REDCap server. Therefore, the risk of loss of data is almost non-existent in the case of a tablet being lost or stolen.
- All data is stored on the REDCap database and backed up on a Wits RHI cloud-based server, both of which can only be accessed with password-protected logins.
- Study statisticians will analyse the data and will not receive any individually identifiable data. Study statisticians only have access to de-identified and de-linked data.
- Data will be de-identified by removing all personally identifying information from the database. If required, all dates will also be perturbed (date shifted) to further preserve the confidentiality of records.
- De-identified electronic data may need to be transferred from the central REDCap database to investigators or statisticians and vice versa. Each investigator will be assigned a secure username and password with appropriate data management privileges to access data via the REDCap online platform. De-identified, password-protected data may be transferred between investigators or statisticians via email, if files are too large for REDCap transfer. Passwords will always be sent in separate emails and will be different for each file transferred.
- All final study databases will not contain any individually identifiable data.
- For any secondary analyses, permission to conduct the analysis must be sought from the Principal Investigators as well as regulatory authorities. No personally identifying information will be shared for secondary analyses – only de-identified, de-linked data.

9.5. Data Analysis

A Study Analysis Plan (SAP)for the study will be developed, reviewed and signed off by all protocol co-chairs. All available data will be included in the analysis. Briefly, the data analysis will include the immunogenicity, reactogenicity and safety analyses described below.

9.5.1. Immunogenicity analyses

For beta and delta variants of concern as well as other emerging variants of concern, we will compute the following:

- Geometric Mean Concentration (GMC) of SARS-CoV-2 serum neutralising antibody levels (using PNA and LVNA assays) and T-cell responses, among Group A participants, at baseline compared to 2-weeks, 3 months and 6-months post J&J Ad26.COV2.S $5x10^{10}$ vp/ml booster vaccination, overall, stratified by PLHIV and HIV-uninfected participants and stratified by participants ≥ 55 years and < 55 years. In addition, data analysis will be conducted at 12-, 18- and 24-month visits on the extension study.
- GMC of SARS-CoV-2 serum neutralising antibody levels (using PNA and LVNA assays) and T-cell responses, among Group B participants, at baseline compared to 2-weeks, 3-months and 6-months post J&J Ad26.COV2.S $2.5x10^{10}$ vp/ml booster vaccination, overall, stratified by PLHIV and HIV-uninfected participants and stratified by participants ≥ 55 years and < 55 years. In addition, data analysis will be conducted at 12-, 18- and 24-month visits on the extension study.
- GMC of SARS-CoV-2 serum neutralising antibody levels (using PNA and LVNA assays) and T-cell responses, among Group C participants, at baseline compared to 2-weeks, 3 months and 6-months post Pfizer BNT162b2 30mcg booster vaccination, overall, stratified by PLHIV and HIV-uninfected participants and stratified by participants ≥ 55 years and < 55 years. In addition, data analysis will be conducted at 12-, 18- and 24-month visits on the extension study.
- GMC of SARS-CoV-2 serum neutralising antibody levels (using PNA and LVNA assays) and T-cell responses, among Group D participants, at baseline compared to 2-weeks, 3-months and 6-months post Pfizer BNT162b2 15mcg booster vaccination, stratified by PLHIV and HIV-uninfected participants and stratified by participants ≥ 55 years and < 55 years. In addition, data analysis will be conducted at 12-, 18- and 24-month visits on the extension study.
- Comparison of GMC of SARS-CoV-2 serum neutralising antibody levels (using PNA and LVNA assays) and T-cell responses, at baseline, 2-weeks, 3-months and 6-months, between Groups A, B, C and D participants. In addition, data analysis will be conducted at 12-, 18- and 24-month visits on the extension study.
- Comparison of GMC of SARS-CoV-2 serum neutralising antibody levels (using PNA and LVNA assays) and T-cell responses, among Group C and D participants, at baseline compared to 2-weeks, 3-months and 6-months post heterologous Pfizer BNT162b2 booster vaccinations, stratified by duration of time between prime and boost (<6 months vs ≥ 6 months) to assess whether duration of time between prime and heterologous boost impacts immunogenicity. In addition, data analysis will be conducted at 12-, 18- and 24-month visits on the extension study.

Non-normally distributed data will be log-transformed prior to analysis. The GMC and associated 95% confidence intervals will be computed for each group at all time points noted above, by computing the anti-log of the mean difference of the log-transformed data. Geometric mean fold rises from baseline and corresponding 95% confidence intervals will be computed. Baseline levels will be established at the enrolment visit. Graphical representations of immunologic parameters will also be produced, as appropriate.

9.5.2. Safety and Reactogenicity analyses

Counts and percentages of solicited, related unsolicited, ≥ grade 3AEs and SAEs will be reported as described above.

10. References

1. Johns Hopkins University of Medicine. Coronavirus Resource Center 2021 [Available from: https://coronavirus.jhu.edu/map.html.
2. CDC. SARS-CoV-2 Variant Classifications and Definitions 2021 [Available from: www.cdc.gov
3. Network for Genomic Surveillance in South Africa. SARS-CoV-2 sequencing update. 2021 [Available from: (https://www.nicd.ac.za/wp-content/uploads/2021/12/Update-of-SA-sequencing-data-from-GISAID-3-Dec-21-Final.pdf).
4. Sheikh A, McMenamin J, Taylor B, Robertson C. SARS-CoV-2 Delta VOC in Scotland: demographics, risk of hospital admission, and vaccine effectiveness. Lancet (London, England). 2021;397(10293):2461-2.
5. Nicola Vousden, Rema Ramakrishnan, Kathryn Bunch, Edward Morris, Nigel Simpson, Christopher Gale, et al. Impact of SARS-CoV-2 variant on the severity of maternal infection and perinatal outcomes: Data from the UK Obstetric Surveillance System national cohort. medRxiv. 2021.
6. Wolter N, Jassat W, Walaza S, Welch R, Moultrie H, Groome M, et al. Early assessment of the clinical severity of the SARS-CoV-2 omicron variant in South Africa: a data linkage study. Lancet (London, England). 2022;399(10323):437-46.
7. Davies M-A, Kassanjee R, Rosseau P, Morden E, Johnson L, Solomon W, et al. Outcomes of laboratory-confirmed SARS-CoV-2 infection in the Omicron-driven fourth wave compared with previous waves in the Western Cape Province, South Africa. medRxiv. 2022:2022.01.12.22269148.
8. Richardson SI, Madzorera VS, Spencer H, Manamela NP, van der Mescht MA, Lambson BE, et al. SARS-CoV-2 Omicron triggers cross-reactive neutralization and Fc effector functions in previously vaccinated, but not unvaccinated individuals. medRxiv. 2022:2022.02.10.22270789.
9. World Health Organization. Status of COVID-19 Vaccines within WHO EUL/PQ evaluation process 2021 [Available from: WHO-2019-nCoV-vaccines-SAGE-recommendation-Ad26.COV2.S-2021.1-eng.pdf

10. Oxford Martin School, University of Oxford, GCDL. Coronavirus (COVID-19) Vaccinations 2021 [Available from: https://ourworldindata.org/covid-vaccinations.
11. Sadoff J, Gray G, Vandebosch A, Cárdenas V, Shukarev G, Grinsztejn B, et al. Safety and Efficacy of Single-Dose Ad26.COV2.S Vaccine against Covid-19. The New England journal of medicine. 2021;384(23):2187-201.
12. Gray G. Unpublished data. 2021.
13. Takuva S, Takalani A, Garrett N, Goga A, Peter J, Louw V, et al. Thromboembolic Events in the South African Ad26.COV2.S Vaccine Study. The New England journal of medicine. 2021;385(6):570-1.
14. Barouch DH, Stephenson KE, Sadoff J, Yu J, Chang A, Gebre M, et al. Durable Humoral and Cellular Immune Responses 8 Months after Ad26.COV2.S Vaccination. The New England journal of medicine. 2021.
15. Sadoff J, Le Gars M, Cardenas V, Shukarev G, Vaissiere N, Heerwegh D, et al. Durability of antibody responses elicited by a single dose of Ad26.COV2.S and substantial increase following late boosting. medRxiv. 2021:2021.08.25.21262569.
16. Khoury DS, Cromer D, Reynaldi A, Schlub TE, Wheatley AK, Juno JA, et al. Neutralizing antibody levels are highly predictive of immune protection from symptomatic SARS-CoV-2 infection. Nature medicine. 2021;27(7):1205-11.
17. Tada T, Zhou H, Samanovic MI, Dcosta BM, Cornelius A, Mulligan MJ, et al. Comparison of Neutralizing Antibody Titers Elicited by mRNA and Adenoviral Vector Vaccine against SARS-CoV-2 Variants. bioRxiv : the preprint server for biology. 2021.
18. Gray GE, Collie S, Garrett N, Goga A, Champion J, Zylstra M, et al. Vaccine effectiveness against hospital admission in South African health care workers who received a homologous booster of Ad26.COV2 during an Omicron COVID19 wave: Preliminary Results of the Sisonke 2 Study. medRxiv. 2021:2021.12.28.21268436.
19. Walsh EE, Frenck RW, Jr., Falsey AR, Kitchin N, Absalon J, Gurtman A, et al. Safety and Immunogenicity of Two RNA-Based Covid-19 Vaccine Candidates. The New England journal of medicine. 2020;383(25):2439-50.

20. Polack FP, Thomas SJ, Kitchin N, Absalon J, Gurtman A, Lockhart S, et al. Safety and Efficacy of the BNT162b2 mRNA Covid-19 Vaccine. The New England journal of medicine. 2020;383(27):2603-15.
21. Statistica. Number of COVID-19 vaccine doses administered in the United States as of September 4, 2021, by vaccine manufacturer 2021 [Available from: https://www.statista.com/statistics/1198516/covid-19-vaccinations-administered-us-by-company/.
22. Gargano JW, Wallace M, Hadler SC, Langley G, Su JR, Oster ME, et al. Use of mRNA COVID-19 Vaccine After Reports of Myocarditis Among Vaccine Recipients: Update from the Advisory Committee on Immunization Practices - United States, June 2021. MMWR Morbidity and mortality weekly report. 2021;70(27):977-82.
23. Allergic Reactions Including Anaphylaxis After Receipt of the First Dose of Pfizer-BioNTech COVID-19 Vaccine - United States, December 14-23, 2020. MMWR Morbidity and mortality weekly report. 2021;70(2):46-51.
24. Pilishvili T, Fleming-Dutra KE, Farrar JL, Gierke R, Mohr NM, Talan DA, et al. Interim Estimates of Vaccine Effectiveness of Pfizer-BioNTech and Moderna COVID-19 Vaccines Among Health Care Personnel - 33 U.S. Sites, January-March 2021. MMWR Morbidity and mortality weekly report. 2021;70(20):753-8.
25. Haas EJ, Angulo FJ, McLaughlin JM, Anis E, Singer SR, Khan F, et al. Impact and effectiveness of mRNA BNT162b2 vaccine against SARS-CoV-2 infections and COVID-19 cases, hospitalisations, and deaths following a nationwide vaccination campaign in Israel: an observational study using national surveillance data. Lancet (London, England). 2021;397(10287):1819-29.
26. Lopez Bernal J, Andrews N, Gower C, Gallagher E, Simmons R, Thelwall S, et al. Effectiveness of Covid-19 Vaccines against the B.1.617.2 (Delta) Variant. The New England journal of medicine. 2021.
27. Collie S, Champion J, Moultrie H, Bekker L-G, Gray G. Effectiveness of BNT162b2 Vaccine against Omicron Variant in South Africa. The New England journal of medicine. 2022;386(5):494-6.
28. Keeton R, Tincho MB, Ngomti A, Baguma R, Benede N, Suzuki A, et al. SARS-CoV-2 spike T cell responses induced upon vaccination or

infection remain robust against Omicron. medRxiv. 2021:2021.12.26.21268380.
29. AVERT. HIV AND AIDS IN SOUTH AFRICA 2021 [Available from: https://www.avert.org/professionals/hiv-around-world/sub-saharan-africa/south-africa.
30. Avelino-Silva VI, Miyaji KT, Hunt PW, Huang Y, Simoes M, Lima SB, et al. CD4/CD8 Ratio and KT Ratio Predict Yellow Fever Vaccine Immunogenicity in HIV-Infected Patients. PLoS neglected tropical diseases. 2016;10(12):e0005219.
31. Carson PJ, Schut RL, Simpson ML, O'Brien J, Janoff EN. Antibody class and subclass responses to pneumococcal polysaccharides following immunization of human immunodeficiency virus-infected patients. The Journal of infectious diseases. 1995;172(2):340-5.
32. Cooper C, Thorne A, Klein M, Conway B, Boivin G, Haase D, et al. Immunogenicity is not improved by increased antigen dose or booster dosing of seasonal influenza vaccine in a randomized trial of HIV infected adults. PloS one. 2011;6(3):e17758.
33. Fuster F, Vargas JI, Jensen D, Sarmiento V, Acuña P, Peirano F, et al. CD4/CD8 ratio as a predictor of the response to HBV vaccination in HIV-positive patients: A prospective cohort study. Vaccine. 2016;34(16):1889-95.
34. Shinde V, Bhikha S, Hoosain Z, Archary M, Bhorat Q, Fairlie L, et al. Efficacy of NVX-CoV2373 Covid-19 Vaccine against the B.1.351 Variant. The New England journal of medicine. 2021;384(20):1899-909.
35. Woldemeskel BA, Karaba AH, Garliss CC, Beck EJ, Wang KH, Laeyendecker O, et al. The BNT162b2 mRNA Vaccine Elicits Robust Humoral and Cellular Immune Responses in People Living with HIV. Clinical Infectious Diseases. 2021.
36. Madhi SA, Koen AL, Izu A, Fairlie L, Cutland CL, Baillie V, et al. Safety and immunogenicity of the ChAdOx1 nCoV-19 (AZD1222) vaccine against SARS-CoV-2 in people living with and without HIV in South Africa: an interim analysis of a randomised, double-blind, placebo-controlled, phase 1B/2A trial. The Lancet HIV. 2021.
37. Choi WS, Cheong HJ. COVID-19 Vaccination for People with Comorbidities. Infect Chemother. 2021;53(1):155-8.

38. Lu S. Heterologous prime-boost vaccination. Current opinion in immunology. 2009;21(3):346-51.
39. Liu X, Shaw RH, Stuart ASV, Greenland M, Aley PK, Andrews NJ, et al. Safety and immunogenicity of heterologous versus homologous prime-boost schedules with an adenoviral vectored and mRNA COVID-19 vaccine (Com-COV): a single-blind, randomised, non-inferiority trial. Lancet (London, England). 2021.
40. Spencer AJ, McKay PF, Belij-Rammerstorfer S, Ulaszewska M, Bissett CD, Hu K, et al. Heterologous vaccination regimens with self-amplifying RNA and adenoviral COVID vaccines induce robust immune responses in mice. Nature communications. 2021;12(1):2893.
41. Borobia AM, Carcas AJ, Pérez-Olmeda M, Castaño L, Bertran MJ, García-Pérez J, et al. Immunogenicity and reactogenicity of BNT162b2 booster in ChAdOx1-S-primed participants (CombiVacS): a multicentre, open-label, randomised, controlled, phase 2 trial. Lancet (London, England). 2021;398(10295):121-30.
42. Shaw RH, Stuart A, Greenland M, Liu X, Nguyen Van-Tam JS, Snape MD. Heterologous prime-boost COVID-19 vaccination: initial reactogenicity data. Lancet (London, England). 2021;397(10289):2043-6.
43. Atmar RL, Lyke KE, Deming ME, Jackson LA, Branche AR, El Sahly HM, et al. Homologous and Heterologous Covid-19 Booster Vaccinations. The New England journal of medicine. 2022:NEJMoa2116414.
44. A Stuart, RH Shaw, X Liu, M Greenland, PK Aley, NJ Andrews, et al. Immunogenicity, safety, and reactogenicity of heterologous COVID-19 primary vaccination incorporating mRNA, viral-vector, and protein-adjuvant vaccines in the UK (Com-COV2): a single-blind, randomised, phase 2, non-inferiority trial. Lancet (London, England). 2022;399(10319):36–49.
45. Chen C, Yang L, Chang W, Huang YC, Chiu CH, Shih SR, et al. A randomized controlled trial of heterologous ChAdOx1 nCoV-19 and recombinant subunit vaccine MVC-COV1901 against COVID-19. Nature communications. 2022;13(1).

46. Chalkias S., Harper C., Vrbicky K., Walsh S. R., Essink B., Brosz A., et al. A Bivalent Omicron-Containing Booster Vaccine against Covid-19. The New England journal of medicine. 2022;387(14):1279–91.
47. Nzolo D, Engo Biongo A, Kuemmerle A, Lusakibanza M, Lula Y, Nsengi N, et al. Safety profile of fractional dosing of the 17DD Yellow Fever Vaccine among males and females: Experience of a community-based pharmacovigilance in Kinshasa, DR Congo. Vaccine. 2018;36(41):6170-82.
48. Casey RM, Harris JB, Ahuka-Mundeke S, Dixon MG, Kizito GM, Nsele PM, et al. Immunogenicity of Fractional-Dose Vaccine during a Yellow Fever Outbreak - Final Report. The New England journal of medicine. 2019;381(5):444-54.
49. Mashunye TR, Ndwandwe DE, Dube KR, Shey M, Shelton M, Wiysonge CS. Fractional dose compared with standard dose inactivated poliovirus vaccine in children: a systematic review and meta-analysis. The Lancet Infectious diseases. 2021;21(8):1161-74.
50. Wibmer CK, Ayres F, Hermanus T, Madzivhandila M, Kgagudi P, Oosthuysen B, et al. SARS-CoV-2 501Y.V2 escapes neutralization by South African COVID-19 donor plasma. Nature medicine. 2021;27(4):622-5.
51. Keeton R, Richardson SI, Moyo-Gwete T, Hermanus T, Tincho MB, Benede N, et al. Prior infection with SARS-CoV-2 boosts and broadens Ad26.COV2.S immunogenicity in a variant dependent manner. medRxiv. 2021:2021.07.24.21261037.
52. Keeton R, Richardson SI, Moyo-Gwete T, Hermanus T, Tincho MB, Benede N, et al. Prior infection with SARS-CoV-2 boosts and broadens Ad26.COV2.S immunogenicity in a variant-dependent manner. Cell Host Microbe. 2021;29(11):1611-9 e5.
53. Keeton R, Tincho MB, Ngomti A, Baguma R, Benede N, Suzuki A, et al. T cell responses to SARS-CoV-2 spike cross-recognize Omicron. Nature. 2022.
54. Riou C, Keeton R, Moyo-Gwete T, Hermanus T, Kgagudi P, Baguma R, et al. Escape from recognition of SARS-CoV-2 variant spike epitopes but overall preservation of T cell immunity. Sci Transl Med. 2022;14(631):eabj6824.

55. US Department of Health and Human Services, Office for Human Research Protections (OHRP). Expedited Review: Categories of Research that may be Reviewed Through an Expedited Review Procedure (1998) 1998 [Available from: https://www.hhs.gov/ohrp/regulations-and-policy/guidance/categories-of-research-expedited-review-procedure-1998/index.html.
56. Greinacher A, Thiele T, Warkentin TE, Weisser K, Kyrle PA, Eichinger S. Thrombotic Thrombocytopenia after ChAdOx1 nCov-19 Vaccination. The New England journal of medicine. 2021;384(22):2092-101.
57. Scully M, Singh D, Lown R, Poles A, Solomon T, Levi M, et al. Pathologic Antibodies to Platelet Factor 4 after ChAdOx1 nCoV-19 Vaccination. The New England journal of medicine. 2021;384(23):2202-11.
58. Moyo-Gwete T, Madzivhandila M, Makhado Z, Ayres F, Mhlanga D, Oosthuysen B, et al. SARS-CoV-2 501Y.V2 (B.1.351) elicits cross-reactive neutralizing antibodies. bioRxiv : the preprint server for biology. 2021.
59. U.S. Department of Health and Human Services, Food and Drug Administration. Guidance for Industry. Toxicity Grading Scale for Healthy Adult and Adolescent Volunteers Enrolled in Preventive Vaccine Clinical Trials. 2007.
60. South African Health Products Regulatory Authority. Safety Reporting During Clinical Trials In South Africa 2019 [Available from: https://www.sahpra.org.za/wp-content/uploads/2020/02/2_Safety-Reporting-during-Clinical-Trial_Nov19_v3-1.pdf.
61. Harris PA, Taylor R, Thielke R, Payne J, Gonzalez N, Conde JG. Research electronic data capture (REDCap)--a metadata-driven methodology and workflow process for providing translational research informatics support. J Biomed Inform. 2009;42(2):377-81.

11. Appendices

1. Brighton Collaboration Case Definition of Anaphylaxis for use in *SPSU* study

2. Toxicity Grading Scale for Healthy Adult and Adolescent Volunteers Enrolled in Preventive Vaccine Clinical Trials, September 2007
