# Supplementary material for: Safety and immunogenicity of booster vaccination and fractional dosing with Ad26.COV2.S or BNT162b2 in Ad26.COV2.S-vaccinated participants
Source: PLOS Glob Public Health. 2024 Apr 11;4(4):e0002703. doi: 10.1371/journal.pgph.0002703 (PMC11008839; doi:10.1371/journal.pgph.0002703)
Supplement: S1 Fig — (DOCX) [file pgph.0002703.s003.docx]

**S1 Fig**

**S1 Figure:** **SARS-CoV-2 spike-specific T cell responses in participants stratified by HIV status in each study arm.** Frequency of spike-specific CD4+ T cells **(A)** and spike-specific CD8+ T cells **(B)** before (BL) and 2 weeks after vaccine boost (W2) in HIV-negative participants and in PLWH. Bars represent medians. Viremic PLWH are identified with a cross. A two-tailed Wilcoxon signed-rank test was used to assess statistical differences between paired samples and a Kruskal-Wallis with Dunn’s corrections was used to compare different groups.
