# Supplementary material for: Safety and immunogenicity of booster vaccination and fractional dosing with Ad26.COV2.S or BNT162b2 in Ad26.COV2.S-vaccinated participants
Source: PLOS Glob Public Health. 2024 Apr 11;4(4):e0002703. doi: 10.1371/journal.pgph.0002703 (PMC11008839; doi:10.1371/journal.pgph.0002703)
Supplement: S2 Table — (DOCX) [file pgph.0002703.s005.docx]

**S2 Table**

|  | Vax-naive | J&J primed | J&J boosted |
| --- | --- | --- | --- |
| N | 14 | 14 | 6 |
| Age* | 46 (37-57) | 46 (37-57) | 55 (49-57) |
| Gender (% female) | 100% | 100% | 100% |
| Time since last vaccination (days)* | na | 44 (31-53) | 110 (96-117) |

**S2 Table:** **Clinical characteristics of samples used for Ad26-specific antibody response assessment.** (Related to Figure 10F). *: Median and Interquartile range (IQR), na: not applicable.
