# Supplementary material for: Safety and immunogenicity of booster vaccination and fractional dosing with Ad26.COV2.S or BNT162b2 in Ad26.COV2.S-vaccinated participants
Source: PLOS Glob Public Health. 2024 Apr 11;4(4):e0002703. doi: 10.1371/journal.pgph.0002703 (PMC11008839; doi:10.1371/journal.pgph.0002703)
Supplement: S3 Table — (DOCX) [file pgph.0002703.s006.docx]

**S3 Table**

|  | Vax-naive | J&J primed |
| --- | --- | --- |
| N | 20 | 11 |
| Age* | 43 (33-53) | 49 (32-53) |
| Gender (% female) | 90% | 81.8% |
| Time since vaccination (days)* | na | 25 (20-45) |

**S3 Table:** **Clinical characteristics of samples used for Ad26-specific T cell response assessment.** (Related to Figures 11D and 11G). All participants were HIV-uninfected. *: Median and Interquartile range (IQR). na: not applicable.
