# Supplementary material for: Safety and immunogenicity of booster vaccination and fractional dosing with Ad26.COV2.S or BNT162b2 in Ad26.COV2.S-vaccinated participants
Source: PLOS Glob Public Health. 2024 Apr 11;4(4):e0002703. doi: 10.1371/journal.pgph.0002703 (PMC11008839; doi:10.1371/journal.pgph.0002703)
Supplement: Attachment — Submitted filename: Response to reviewers_PGPH-D-23-02273.docx [file pgph.0002703.s007.docx]

**Response to reviewers**

PGPH-D-23-02273

Reviewer #1:

 Riou et al in the manuscript “Safety and immunogenicity of booster vaccination and fractional dosing with Ad26.COV2.S or BNT162b2 in Ad26.COV2.S-vaccinated participants” investigated the antibody and T-cell responses upon either a full or half-dose booster of Ad26.COV2.S or BNT162b2 vaccine. They noted an increased immunogenicity after heterologous immunization compared with the homologous regimen. This study is rationally designed and the provided data justify the overall conclusion.

I agree with what the authors stated “…the almost complete absence of boosting by this vaccine (Ad26.Cov2.S) in our cohort was striking” (page 25, lines 638-639). Moreover, I think the Ad26 booster data overall is in discordance with the previous studies in humans (e.g., Interim Results of a Phase 1–2a Trial of Ad26.COV2.S Covid-19 Vaccine, Sadoff et al. NEJM 2021, and Immunogenicity of the Ad26.COV2.S Vaccine for COVID-19, Stephenson et al. JAMA 2021) and in macaques (A homologous or variant booster vaccine after Ad26.COV2.S immunization enhances SARS-CoV-2–specific immune responses in rhesus macaques, He et al. Sci Trans Med, 2022). Can the authors provide comments on that?

>> We thank the reviewer for bringing up this important point. We agree with the reviewer that some studies have shown a significant increase (approximately 2 to 3-fold) in spike-specific IgG, neutralizing antibodies, and spike-specific T cell responses following a homologous Ad26.COV2.S boosting. However, both our data and results from other research groups [Khoo, 2022, Med, 10.1016/j.medj.2021.12.004; Geers, 2022, iScience, 10.1016/j.isci.2022.105753] indicate a more limited, if any, boosting of the immune response with a homologous Ad26.COV2.S boost. These discrepancies in the magnitude of homologous boosting may be attributed to several factors, including: 1) the participants previous history of SARS-CoV-2 infection. Notably, in the studies referenced by the reviewer, participants were all SARS-CoV-2 naïve, whereas in our study, more than 80% had a history of prior SARS-CoV-2 infection, 2) the timing between dose administration and 3) pre-existing or vaccine-induced anti-vector immunity. While direct associations between anti-vector immunity and Ad26.COV2.S response were not evident in our results, it is clear that Ad26.COV2.S vaccination (prime or boost) increases anti-vector antibody and T cell responses. Further analysis is warranted to fully assess whether anti-vector immunity could potentially interfere with the Ad26.COV2.S response.

Regarding the effect of homologous versus heterologous boosting, recent publications consistently demonstrate that heterologous regimens containing mRNA-based vaccines exhibit superior potency in enhancing both humoral and cellular immune responses compared to homologous boosting [Atmar, 2022, NEJM, 10. 1056/NEJMoa2116414 ; Sablerolles, 2022, NEJM, 10.1056/NEJMoa2116747 ; Geers, 2022, iScience, 10.1016/j.isci.2022.105753 ; Stuart, 2022, Lancet, 10.1016/S0140-6736(21)02718-5 ; Khoo, 2022, Med, 10.1016/j.medj.2021.12.004]. The superiority of heterologous vaccination (Ad26.COV2.S followed by BNT162b2) could be related to its ability to broaden the humoral and cellular spike-specific repertoire, expanding antibodies and T cells capable of recognizing the S2 region of Spike. Moreover, a vaccine efficacy study showed that vaccine efficacy was highest for regimens incorporating a booster dose of an mRNA vaccine and lowest for the homologous Ad26.COV2.S/Ad26.COV2.S regimen [Accorsi, 2022, NEJM, 10.1056/NEJMc2203165].

Overall, our results align with prior research, indicating that heterologous vaccination transiently boosts spike-specific humoral and cellular immunity in individuals vaccinated with Ad26.COV2.S. Conversely, the impact of a homologous boost with Ad26.COV2.S was found to be modest compared to heterologous regimen.

To clarify the first point, some text has been added to the discussion and reads as follow:

**Page 23, Line 597-610**: “Conversely, a homologous Ad26.COV2.S boost yielded mixed results. While some studies have demonstrated a significant increase (approximately 2- to 3-fold) in spike-specific IgG, neutralizing antibodies, and spike-specific T cell responses, irrespective of the dosage administered (Sadoff, 2021; Stephenson, 2021), others have reported more modest effects, if any, in enhancing these immune responses (Khoo, 2022; Sablerolles, 2022; Atmar, 2022; Geers, 2023). These differences could be related to several factors, including an individual’s previous history of SARS-CoV-2 infection, the interval between doses, the timing between doses and/or the potential impact of pre-existing or vaccine-induced anti-vector immunity.

The superiority of heterologous vaccination over homologous vaccination is reinforced by a vaccine efficacy study showing that vaccine efficacy was highest for regimens incorporating a booster dose of an mRNA vaccine and lowest for the Ad26.COV2.S/Ad26.COV2.S regimen (Accorsi, 2022). This could be related to the ability of heterologous vaccination (Ad26.COV2.S followed by BNT162b2) to broaden the humoral and cellular spike-specific repertoire, thereby expanding antibodies and T cells capable of recognizing the S2 region of Spike (Khoo, 2022).

We have also moderated our statement that was on page 25, lines 638-639).

**Page 25, Line 651:** “Although the Ad26.COV2.S is known to trigger lower responses than BNT162b2, the limited effect observed after homologous boosting in our cohort was striking.”

**Editors**

As requested, author initials have been added for each funding source and the role of funders has been stated in the acknowledgement section (as follow: “The funders had no role in study design, data collection and analysis, decision to publish, or preparation of the manuscript”).

**Please see page 28, line 701.**

All data underlying the findings described in this manuscript are freely available to other researchers. They have included in the supplementary material as supp table (excel format).

**Please see page 12, line 325:** “All data underlying the findings described in this manuscript are available in supplementary table 1.”
